# The effects of total sleep deprivation on the circadian rhythms and psychophysiological factors in military cadets; a comparison between wakefulness in light and darkness

**DOI:** 10.3389/fphys.2025.1732257

**Published:** 2026-01-16

**Authors:** Kateřina Skálová, Jan Maleček, David Kolář, Kateřina Červená, Jana Kopřivová, James Tufano, Dan Omcirk, Jan Padecký, Tomas Vetrovsky, Zdeňka Bendová

**Affiliations:** 1 Sleep and Chronobiology Research Center, National Institute of Mental Health, Klecany, Czechia; 2 Faculty of Science, Charles University, Prague, Czechia; 3 Faculty of Physical Education and Sport, Charles University, Prague, Czechia; 4 Third Faculty of Medicine, Charles University, Prague, Czechia

**Keywords:** circadian clock, cognition, light at night, melatonin, sleep deprivation, taste preference

## Abstract

**Objectives:**

Both sleep deprivation (SD) and light at night have negative effects on human health and performance. The aim of our work was to compare the intermediate effects of total SD under two lighting conditions: full indoor lighting and darkness mimicking natural nocturnal wakefulness.

**Methods:**

We examined melatonin levels during SD nights, locomotor activity and peripheral temperature rhythms, cognitive performance, mood, hunger, glycaemia and food preference after SD and recovery sleep. Statistical evaluation included ANOVA with FDR correction and confidence intervals.

**Results:**

SD transiently altered peripheral temperature rhythm and post-SD activity, with faster resynchronisation after SD in darkness. Subjective sleepiness increased after SD, with light at night alleviating morning sleepiness. Positive affect decreased after SD but normalised after recovery sleep in both groups. Negative affect worsened in the morning after SD in darkness. Cognitive performance declined after SD, but this effect was higher after SD in darkness. Preprandial glycaemia was higher after recovery sleep following SD in darkness, and sweet taste preference was significantly higher after SD in darkness.

**Conclusion:**

Light exposure during SD may lead to lower subjective sleepiness and better cognitive performance the next morning compared to SD in darkness. However, light during SD also causes more pronounced and persistent disruptions to circadian rhythms of temperature and activity. This underscores the trade-off between the short-term benefits of nocturnal light exposure and its potential long-term impacts on circadian health.

## 1 Introduction

Total sleep deprivation (SD) significantly increases the risk of accidents by impairing cognitive functions such as alertness, attention, memory, reaction time, and decision-making leading to errors in activities like driving and operating machinery. It also disrupts mood, socio-emotional functioning (Groeger et al., 2022) hunger, appetite, and subjective food preferences (Liu et al., 2022).

SD arises from factors like work demands, lifestyle choices, environmental conditions, chronic medical issues (e.g., persistent pain or diabetes), and mental health conditions such as chronic stress or depression (Liew and Aung, 2021). Previous work has shown that morning alertness can be affected by modifiable factors such as prior physical activity and meal composition (Vallat et al., 2022). However, a notable distinction lies in the presence or absence of nocturnal light. Sleep deprivation in illuminated environments, such as in a hospital or care facility, may have distinct physiological and behavioural consequences compared to wakefulness in darkness.

Research in recent decades has focused on the effects of nocturnal light on the circadian system. Disrupted circadian function is associated with metabolic disorders, cardiovascular diseases, and impaired mental health (Walker et al., 2020; Meléndez-Fernández et al., 2023). Laboratory studies show that light, particularly with high blue spectral content, delays sleep onset, enhances attention, and affects cognitive performance (Gumenyuk et al., 2012; Šmotek et al., 2020; Cajochen et al., 2022; Sunde et al., 2022). A specific effect of nocturnal light, but not SD, is the suppression of pineal melatonin synthesis, regulated by the suprachiasmatic nucleus (SCN) to occur at night and in darkness. Once produced, melatonin circulates via the bloodstream and cerebrospinal fluid, aiding circadian rhythm synchronization with the solar cycle (Amaral and Cipolla-Neto, 2018). SD does not directly suppress melatonin production, but light exposure at night, especially with greater melanopic efficacy, significantly suppresses melatonin synthesis within minutes (Gooley et al., 2011). Typical indoor lighting (>300 lux) and even much dimmer light (∼6 lux) can suppress melatonin in some individuals (Phillips et al., 2019; Cain et al., 2020).

Both SD and circadian disruption can influence thermoregulation and activity patterns, typically assessed non-invasively via actigraphy and distal skin temperature, which reflect circadian phase and sleep–wake stability (van Marken Lichtenbelt et al., 2006; Kräuchi, 2007). In addition, both conditions impact metabolic function and food-related behaviour. Experimental studies show that total or partial SD increases hunger and preference for calorie-rich, sweet foods (Benedict et al., 2012; Greer et al., 2013), while circadian misalignment alters glucose metabolism and subjective appetite (Scheer et al., 2009; McHill et al., 2022). Since exogenous melatonin may reduces appetite under nocturnal light exposure (Albreiki et al., 2022), we hypothesised that light during SD may similarly modulate post-deprivation food preferences.

Although light at night and SD often co-occur, their physiological effects are rarely disentangled. Most studies on SD are conducted under light, while circadian studies on nocturnal light usually involve partial or total sleep loss. This overlap complicates interpretation and may obscure distinct effects on circadian regulation, cognition, and behaviour. To address this, we compared the immediate and medium-term effects of total SD under either indoor-like lighting or darkness. We evaluated cognitive performance, mood, hunger, and food preferences following SD and after recovery sleep (RS). Actigraphy was used to assess changes in movement activity days before and after SD, alongside peripheral temperature rhythm profiles. A homogenous cohort of military cadets allowed us to minimise interindividual variability in age, body composition, and general health status—factors that often confound findings in sleep and circadian research in humans.

## 2 Participants and methods

### 2.1 Participants

Eighteen healthy male military cadets (Military Department of Charles University) participated in the study (age 24.1 ± 3.0 years, height 181.5 ± 6.3 cm, weight 79.3 ± 8.3 kg). All were enrolled in Master’s or early-stage PhD programmes and had no prior operational service. Inclusion criteria required no diagnosed psychiatric or neurological disorders, no sleep-affecting medications, and no shift work for at least 1 year. Participants followed a shared daily schedule, resulting in a consistent sleep–wake routine across the cohort. All 18 volunteers underwent sleep deprivation under light conditions (SD/L group), and 12 also participated in sleep deprivation under constant darkness (SD/D group; all abbreviations are defined in Table 1). The Ethics Committee of the National Institute of Mental Health in the Czech Republic (ref. 176/20) approved the study, adhering to ethical standards. Written informed consent was obtained following the Declaration of Helsinki.

**TABLE 1:** Abbreviations and their definitions used in the study. This table provides a comprehensive list of abbreviations used throughout the manuscript, arranged in alphabetical order alongside their corresponding definitions.

Abbreviation	Definition
BD	Before deprivation
BE	Before Sleep deprivation in the evening
BE/D	Before Sleep deprivation in the evening - dark conditions
BE/L	Before Sleep deprivation in the evening - light conditions
BM	Before Sleep deprivation in the morning
BM/D	Before Sleep deprivation in the morning - dark conditions
BM/L	Before Sleep deprivation in the morning - light conditions
D1-D5	First day after SD (D1) and the following days (D2-D5)
D1AB/D	D1 after breakfast - dark conditions
D1AB/L	D1 after breakfast - light conditions
D1AD/D	D1 after dinner - dark conditions
D1AD/L	D1 after dinner - light conditions
D1AL/D	D1 after lunch - dark conditions
D1AL/L	D1 after lunch - light conditions
D1BB/D	D1 before breakfast - dark conditions
D1BB/L	D1 before breakfast - light conditions
D1BD/D	D1 before dinner - dark conditions
D1BD/L	D1 before dinner - light conditions
D1BL/D	D1 before lunch - dark conditions
D1BL/L	D1 before lunch - light conditions
D1E	D1 in the evening
D1E/D	D1 in the evening - dark conditions
D1E/L	D1 in the evening - light conditions
D1M	D1 in the morning
D1M/D	D1 in the morning - dark conditions
D1M/L	D1 in the morning - light conditions
IV	Intra-daily variability
L5	The least active five-hour period
M10	The most active ten-hour period
Mo/AD	Monday after Sleep deprivation
Mo/AD/D	Monday after Sleep deprivation - dark conditions
Mo/AD/L	Monday after Sleep deprivation - light conditions
Mo/BD	Monday before Sleep deprivation
R	Recovery phase, after recovery sleep
RA	Relative amplitude
RAB/D	Recovery day after breakfast - light conditions
RAB/L	Recovery day after breakfast - dark conditions
RBB/D	Recovery day phase before breakfast - dark conditions
RBB/L	Recovery day phase before breakfast - light conditions
RM	Morning after recovery sleep
RM/D	Morning after recovery sleep - dark conditions
RM/L	Morning after recovery sleep - light conditions
SD/D	Sleep deprivation - dark conditions
SD/L	Sleep deprivation - light conditions

### 2.2 Experimental design

**FIGURE 1:**
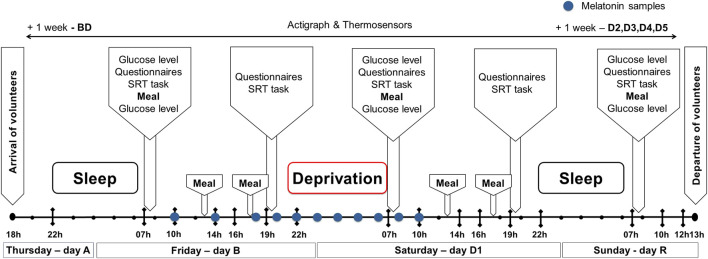
Schematic overview of the experimental design. Participants arrived on Thursday at 6 p.m. and departed on Sunday at 1 p.m. ● = 2-h interval. Only scheduled personalized rations were provided throughout the protocol. SRT, Simple Response Time task; BD, days before deprivation; D1–D5, days after deprivation; R, recovery day.

We conducted a repeated-measures study at the National Institute of Mental Health’s sleep laboratory to assess the effects of ∼39 h of total SD. The study had two experimental phases (February and November) involving SD/L and SD/D groups, respectively, each lasting 4 days (Figure 1).

One week before the start of the protocol, participants were familiarized at the Faculty of Physical Education and Sports, where they learned about the study design, tests, and ethics, and provided informed consent. They were instructed to avoid alcohol for 1 week and caffeine for 48 h before each phase. Physical behaviour and peripheral temperature were monitored using actigraphs and sensors, starting 1 week before and continuing 1 week after each phase.

Subjects arrived at the sleep laboratory on Thursday (day A) at ∼6 p.m., completed health questionnaires, and familiarized themselves with the lab and test protocols. They received task instructions and practiced to minimise learning effects. Baseline testing began with uninterrupted sleep (10:00 p.m. to 6:30 a.m.). On Friday (day B, before SD), participants underwent morning testing (7:30–9:30 a.m.) and evening testing (7:00–9:00 p.m.), with saliva sampling from 10 a.m. on Friday to Saturday after SD (Figure 1).

During the SD night (Friday–Saturday), the SD/L group was exposed to constant LED light (∼2700 K; ∼500 lux; ∼240 melanopic lux, measured in the horizontal plane), engaging in passive activities such as board games, TV, and reading in a common room. The SD/D group remained in darkness, occasionally using red headlamps (640 nm, 0.8–1 W/m^2^) while conversing and listening to audio. Both sessions were supervised to prevent napping or daytime sleep, and participants remained on the institution’s premises throughout the day.

On Saturday (day D1), participants completed two test sessions, identical to Friday’s. Recovery sleep occurred on Saturday night (∼10:00 p.m. to spontaneous awakening on Sunday). Final tests were conducted Sunday morning before participants departed with actigraphs and sensors to continue monitoring for 1 week.

Throught the entire study protocol, participants received personalized daily rations of standard Czech military “ready-to-eat” meals. No additional food intake was allowed. Body composition was measured 1 week before the experiment using air displacement plethysmography (Bod Pod Body Composition System; Life Measurement Instruments, Concord, CA). Total daily energy expenditure was calculated based on resting metabolic rate and an “active” physical activity factor of 1.6 (Conkright et al., 2021). Water was consumed *ad libitum*, with meals scheduled at ∼9:30 a.m., ∼12:30 p.m., and ∼5:30 p.m. daily. The experiment was conducted in a standardised hospital environment with regulated temperature and ventilation in compliance with institutional standards. The sleep laboratory is specifically adapted for chronobiological experiments: all windows are fully sealed to prevent any intrusion of outdoor light, no indicator lights are present in the rooms, and the bathroom is equipped only with dim red illumination, the use of which is monitored. Participants wore their own sleepwear or athletic clothing, and no specific control over clothing layers was applied.

### 2.3 Melatonin assay

Saliva samples were collected via passive drool on Friday before SD at 10 a.m. and subsequently at 2 p.m., 6 p.m., 8 p.m., 10 p.m., 12 a.m., 2 a.m., 4 a.m., 6 a.m., 8 a.m., and 10 a.m. the next day. Samples were stored at −80 °C and analyzed in duplicate using double antibody RIA kits (Melatonin direct Serum/Plasma/Saliva RIA, IBL International GmbH) following the manufacturer’s protocol. The kit’s analytical sensitivity was 0.3 pg/mL for saliva, with results expressed in pg/mL.

### 2.4 Peripheral skin temperature measurement

Wrist temperature was recorded every 15 min using iButton DS1921H-F5 sensors (Maxim Integrated, United States), placed on the non-dominant wrist above the radial artery and secured in cotton sweatbands for optimal contact. Participants wore the sensors continuously, except while bathing, for 2 weeks. Data were downloaded via DS1402D-DR8 adapters (IDC, Spain) and analyzed using iButton Viewer v.3.22. Values below 30 °C were excluded. A 24-h cosine function was fitted to the raw data, and averages from 5 days pre- and post-SD characterized circadian profiles and their return to baseline.

### 2.5 Actigraphy

Activity/rest cycles were monitored using MotionWatch 8 actigraphs (Cambridge, Neurotechnology Ltd, UK) on the non-dominant hand for 2 weeks. Data were analyzed with MotionWare software. A 24-h cosine function was fitted to the raw data, and averages from 5 days pre- and post-SD characterized circadian profiles and their return to baseline.

Non-Parametric Circadian Rhythm Analysis (NPCRA) assessed the least active 5-h (L5) and most active 10-h periods (M10), relative amplitude (RA; 0–1), and intra-daily variability (IV; 0–2) for the Monday before and after the SD. Higher RA indicates greater amplitude, while higher IV reflects greater fragmentation.

### 2.6 Simple response time task (SRT)

Participants completed the SRT using the PEBL program, responding to visual stimuli by pressing the space bar. Stimulus timing ranged from 2 to 12 s. Sessions lasted 10 min, conducted twice daily (Figure 1). Reaction times were expressed as the session median for analysis.

### 2.7 Questionnaires

The questionnaire battery included.

#### 2.7.1 Morningness-eveningness questionnaire (MEQ)

Evaluates circadian phenotype through 19 items, categorizing chronotypes from extreme morning (70–86) to extreme evening (16–30) (Horne and Ostberg, 1976).

#### 2.7.2 Stanford sleepiness scale (SSS)

Single-item scale rating sleepiness from one to 7 (Hoddes et al., 1973).

#### 2.7.3 Positive and negative affect schedule (PANAS)

Assesses emotional states through 20 descriptors rated on a Likert scale (1 = very slightly or not at all, 5 = extremely) (Watson et al., 1988). Administered at 10 a.m. and 10 p.m. before and after SD and post-recovery sleep (Figure 1).

#### 2.7.4 Visual analogue scales (VAS) for assessment of appetite sensations

Evaluates hunger and appetite, with responses recorded on a 100 mm scale, measured before and after meals within a 5-min window (Flint et al., 2000). To obtain the resulting score, the distance between the left end and the participant’s marker was determined.

### 2.8 Blood glucose measurement

Glucose levels were measured using the FreeStyle Optium Neo meter (range: 1.1–27.8 mmol/L; ±0.2 mmol/L or ±2%). Measurements were taken before meal and 30 min postprandially to capture glucose response peaks while maintaining experimental continuity (Figure 1).

### 2.9 Data processing

The circadian rhythmicity of melatonin profile, peripheral temperature and activity was analysed using cosinor analysis defined by the equation: [Y = mesor + (amplitude*cos (2π*(X-acrophase)/period] with a fixed period of 24 h. Cosinor p-values correspond to the zero-amplitude test, comparing a 24-h cosine model with a horizontal line to confirm the presence of a rhythm. Parameters of the cosine curve, mesor (the mid-value of the cosine curve, representing a rhythm-adjusted mean), amplitude (the difference between the peak or trough and the mean value of the cosine curve), and acrophase (the time of peak value of the fitted curve, representing the average time of high values in the data), were calculated.

### 2.10 Statistics

Diurnal melatonin secretion profiles were analysed using two-way repeated measures ANOVA (RM ANOVA), with time and condition as factors. Circadian profiles of body temperature and activity were analysed using one-way RM ANOVA followed by multiple comparisons with BD as the reference group. Mesor, cosine amplitude and acrophase were compared by one-way ANOVA with Tukey´s multiple comparisons test. Data from NPCRA (L5, M10, RA, IV), SRT, questionnaires, and glycaemia were analysed by RM one-way ANOVA, with participant numbers adjusted to available complete datasets for each condition (18 in SD/L, 12 in SD/D); direct SD/L vs. SD/D comparisons were based on the 12 participants who completed both conditions. All analyses were supported by normality checks and non-parametric confirmation. Effect size was expressed as *R* ^2^, with values interpreted according to Cohen’s guidelines: small effect (*R* ^2^ ≈ 0.01–0.059), medium effect (*R* ^2^ ≈ 0.06–0.137), and large effect (*R* ^2^ ≥ 0.14). Ninety-five percent confidence intervals (CI) for mean differences were obtained from the output of the uncorrected Fisher’s LSD test; CIs not crossing zero were considered to indicate statistical significance. The significance threshold for all ANOVA analyses was set at p < 0.05. In all ANOVA models involving both SD/L and SD/D groups, session or time of day was treated as a within-subject factor, and lighting condition as a between-subject factor. In analyses limited to a single group (e.g., circadian profiles), only the within-subject factor (day or time) was included. All analyses were followed by false discovery rate (FDR) correction using the Benjamini–Hochberg method with q = 0.1 as the threshold for statistical significance. All statistical analyses were performed using GraphPad Prism 10.4.1.

## 3 Results

We aimed to compare the immediate and medium-term effects of SD under light and dark conditions. To support this comparison, we structured the Results section into three domains: circadian rhythms, cognitive performance, and metabolism/food intake.

### 3.1 Effect of lighting regime during SD on circadian rhythms

The average MEQ score (53) indicated an intermediate chronotype, ranging from 33 (one moderate evening chronotype) to 63 (five moderate morning chronotypes).

**FIGURE 2:**
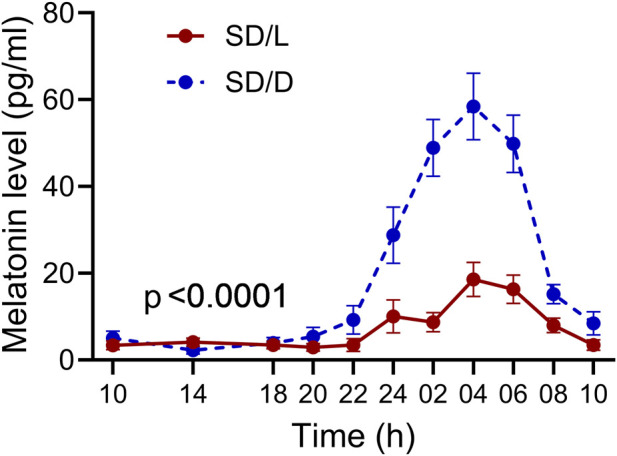
The impact of SD and lighting conditions during SD on the diurnal rhythm of salivary melatonin levels. Each point represents the mean of 12 values ±standard deviation, with p-values indicating the results of the RM two-way ANOVA.

Melatonin secretion, highly sensitive to light, validated our lighting conditions during SD. Figure 2 illustrates the mean profiles for SD/L and SD/D groups. Cosinor model fit confirmed a statistically significant 24-h rhythmicity for both profiles (p < 0.0001). Cosinor amplitudes were higher in SD/D (SD/L: = 6.535; SD/D: = 28.35). The effect of light condition accounted for 13.9% of the total variance (RM two-way ANOVA), which corresponds to a medium-to-large effect size. The between-group difference was statistically significant (F (10, 160) = 13.53; p < 0.0001; 95% CI of difference: −19.44 to −8.42).

**FIGURE 3:**
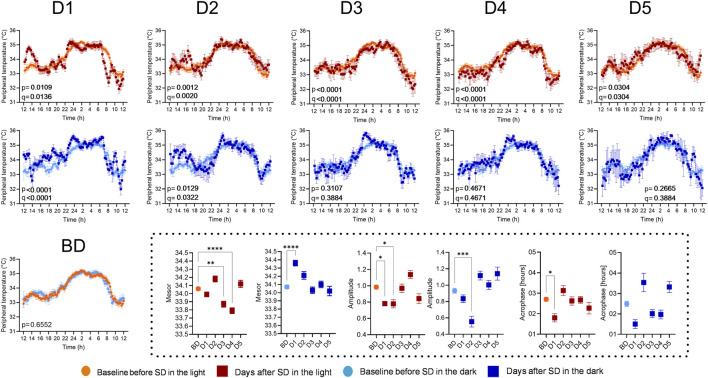
The impact of SD and lighting conditions during SD on the diurnal rhythm of wrist skin temperature. The temperature values measured 5 days before SD were averaged for the groups SD/L (BD, light orange lines and symbols) and SD/D (light blue lines and symbols) and compared with the averaged temperature values for all participants measured on the first day after SD (D1, red or dark blue lines and symbols) and on the subsequent days (D2-D5). Each point represents the mean of 12 values ±standard deviation. p-values indicate the results of RM one-way ANOVA; q-values indicate significance according to the Benjamini–Hochberg FDR correction. Inset: Parameters of circadian rhythms (mesor, amplitude, and acrophase) were compared. Asterisks indicate significance according to one-way ANOVA with Tukey’s multiple comparisons test.

Wrist skin temperature exhibited a circadian rhythm in both SD/L and SD/D groups (cosinor model fit, p < 0.0001). A one-way RM ANOVA (time × condition) revealed a significant interaction effect (SD/L: F (2.869, 272.6) = 18.65, p < 0.0001, *R* ^2^ = 0.1641; SD/D: F (3,546, 333,3) = 10.71, p < 0.0001, *R* ^2^ = 0.1023). Baseline profiles showed no significant differences (Figure 3, panel BD). In the SD/L group, temperature profiles differed from baseline until day 5, whereas in SD/D, profiles aligned with baseline by day 3 (full statistics, including mean differences, 95% CI, uncorrected p-values and FDR-adjusted q-values, are provided in Supplementary Table S1). One-way ANOVA with Tukey’s multiple comparisons test revealed that mesor variations were more prominent than amplitude or acrophase changes (Figure 3, insets; full statistics in Supplementary Table S2).

**FIGURE 4:**
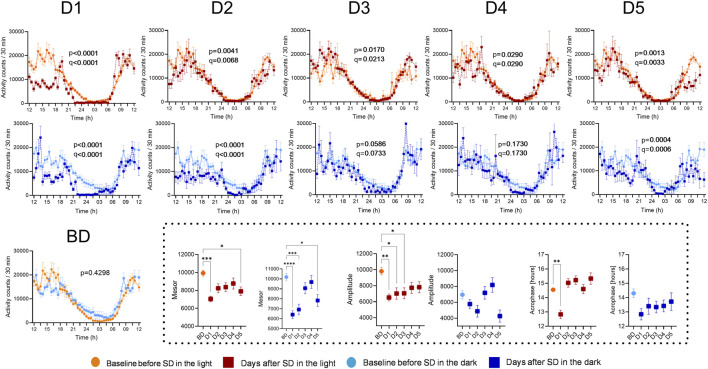
The impact of SD and lighting conditions during SD on the diurnal rhythm of activity. The activity values measured 5 days before SD were averaged for the groups SD/L (BD, light orange lines and symbols) and SD/D (light blue lines and symbols) and compared with the averaged values for all participants measured on the first day after SD (D1, red or dark blue lines and symbols) and on the subsequent days (D2-D5). Each point represents the mean of 12 values ±standard deviation. p-values indicate the results of RM one-way ANOVA; q-values indicate significance according to the Benjamini–Hochberg FDR correction. Inset: Parameters of circadian rhythms (mesor, amplitude, and acrophase) were compared. Asterisks indicates significance according to one-way ANOVA with Tukey’s multiple comparison test.

Actigraphy confirmed circadian rhythms in both groups (cosinor model fit: p < 0.0001) with early afternoon acrophases. A one-way RM ANOVA (time × condition) revealed a significant interaction effect (SD/L: F (2,876, 135,2) = 5.328, p = 0.0020, *R* ^2^ = 0.7972; SD/D: F (3,900, 183,3) = 10.95, p < 0.0001, *R* ^2^ = 0.6896). Baseline activity profiles were similar (Figure 4, panel BD). RM one-way ANOVA detected differences in SD/L profiles until day 5; in SD/D, profiles aligned with baseline by days 3–4 (full statistics, including mean differences, 95% CI, uncorrected p-values and FDR-adjusted q-values, are provided in Supplementary Table S3). Both groups showed lower mesors post-SD, with reduced amplitudes observed only in SD/L during the first 3 days. A phase delay in acrophase occurred solely on D1 in SD/L (Figure 4, insets; full statistics in Supplementary Table S4).

**FIGURE 5:**
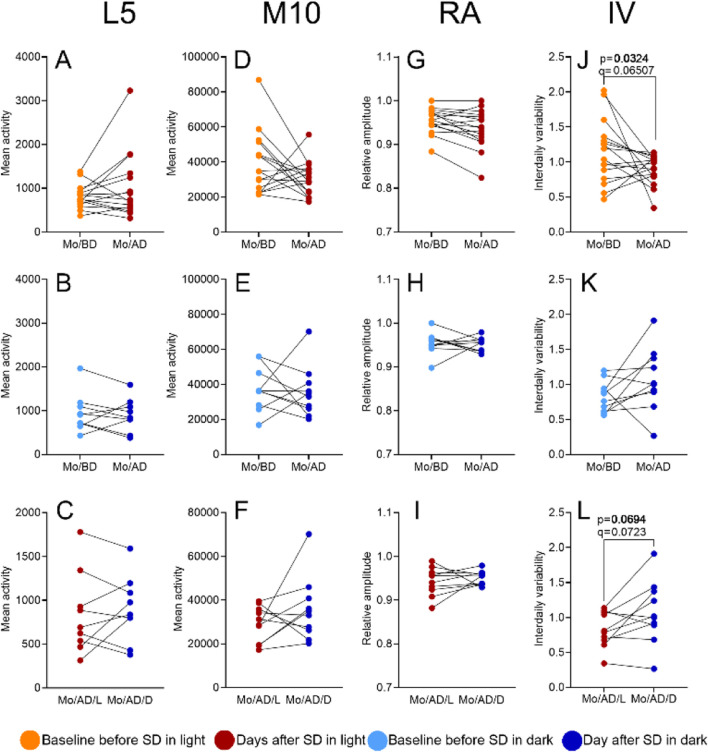
The impact of SD and lighting conditions during SD on the NPCRA variables. Values for the average activity levels during the least active 5-h period (L5; panels **A, B, C**), the most active 10-h period (M10; panels **D, E, F**), the relative amplitude (RA; panels **G, H, I**), and intra-daily variability (IV; panels **J, K, L**) were compared between Monday before SD (Mo/BD) and Monday after SD (Mo/AD) using RM one-way ANOVA. See Materials and Methods for details. p-values refer to the uncorrected results of the RM one-way ANOVA; q-values indicate significance after Benjamini–Hochberg FDR correction.

The parameters obtained from nonparametric circadian rhythm analysis were compared between baseline Mondays (Mo/BD) and post-SD Monday (Mo/AD) using RM one-way ANOVA. Figures 5A,B shows no significant differences in L5 (RM one-way ANOVA: F (2.640, 21.12) = 0.4632, p = 0.6875, *R* ^2^ = 0.055), M10 (F (1,749, 15,74) = 1.429, p = 0.2668, *R* ^2^ = 0.1370; Figures 5D,E), or RA (F (2,321, 20,89) = 0.8330, p = 0.4644; *R* ^2^ = 0.0847; Figures 5G,H) but a difference in IV values (F (1,979, 17,81) = 3.541, p = 0.0477, *R* ^2^ = 0.2896; Figures 5J,K). Post-hoc analysis with Benjamini–Hochberg FDR correction revealed a significant difference in IV between Mo/BD and Mo/AD in the SD/L group (95% CI [0.0644, 0.8664], p = 0.0324, q = 0.0651; *R* ^2^ = 0.290; Figure 5J). The trend towards significance for IV on post-SD Monday (Mo/AD) between groups (95% CI [0.04873, 0.8897], p = 0.0694, q = 0.0723) likely reflects pre-existing disparities in baseline IV values between the SD/L and SD/D groups (Figures 5C,F,I,L; full statistics in Supplementary Table S5).

### 3.2 Effect of lighting regime during SD on cognitive parameters

**FIGURE 6:**
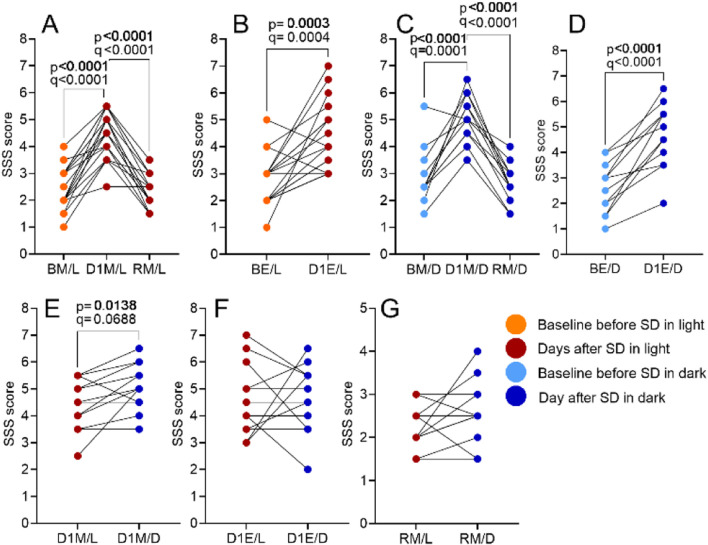
The impact of SD and lighting conditions during SD on the subjective sleepiness score. Scores were compared for the morning before SD (BM), the morning after SD (D1M) and the morning after recovery sleep (RM; panels **A, C**) as well as for the evening before SD (BE) and the evening after SD (D1E; panels **B, D**), using RM one-way ANOVA. Between-session comparisons of corresponding time points are shown in panels **E–G**. See Materials and Methods for details. p-values refer to the uncorrected results of the RM one-way ANOVA; q-values indicate significance after Benjamini–Hochberg FDR correction.

Subjective sleepiness, assessed via SSS, increased significantly after SD nights in both SD/L (F (2.769, 47.07) = 32.21, p < 0.0001, *R* ^2^ = 0.6546; Figures 6A,B) and SD/D (F (1.876, 20.64) = 25.13, p < 0.0001, *R* ^2^ = 0.6955; Figures 6C,D) sessions. In both sessions, sleepiness was higher compared to baseline and recovery day in the morning and evening (Figures 6A,C). Between-session analysis also showed a significant difference (F (4.219, 46.41) = 22.00, p < 0.0001, *R* ^2^ = 0.6667; Figures 6E,E,G). Differences between February and November (BM/L vs. BM/D) were not statistically significant (Supplementary Table S6B). Sleepiness levels were higher in the SD/D group during the morning after SD (Figure 6E; full statistics, including mean differences, 95% CI, uncorrected p-values and FDR-adjusted q-values, are provided in Supplementary Table S6).

**FIGURE 7:**
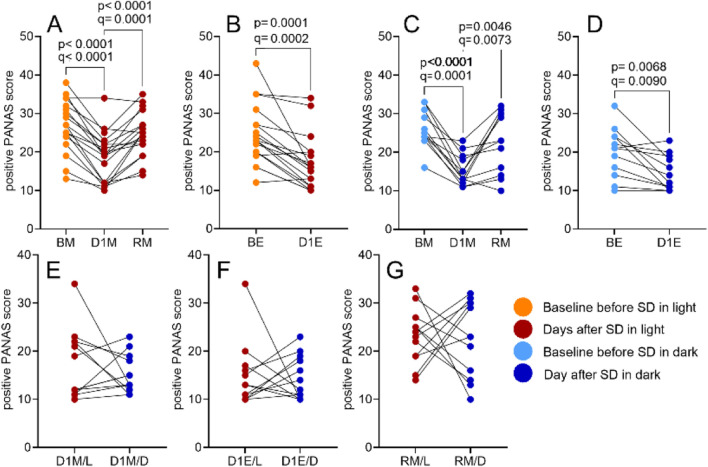
The impact of SD and lighting conditions during SD on the positive PANAS score Scores were compared for the morning before SD (BM), the morning after SD (D1M), and the morning after recovery sleep (RM; panels **A, C**), as well as for the evening before SD (BE) and the evening after SD (D1E; panels **B, D**), using RM one-way ANOVA. Between-session comparisons of corresponding time points are shown in panels **E–G**. See Materials and Methods for details. p-values refer to the uncorrected results of the RM one-way ANOVA; q-values indicate significance after Benjamini–Hochberg FDR correction.

PANAS scores showed a significant decrease in positive affect post-SD in both SD/L (F (3.089, 52.52) = 23.70, p < 0.0001, *R* ^2^ = 0.5823; Figures 7A,B) and SD/D (F (2.346, 25.81) = 12.34, p < 0.0001, *R* ^2^ = 0.5287; Figures 7C,D) sessions returning to baseline after recovery sleep (Figures 7A,C). Between-session analysis also showed a significant difference (F (2.666, 29.33) = 7.404, p = 0.0011, *R* ^2^ = 0.4023), however, no baseline differences (BM/L vs. BM/D) or group differences (SD/L vs. SD/D on D1) were observed with Benjamini–Hochberg FDR correction (Figures 7E–G; full statistics, including mean differences, 95% CI, uncorrected p-values, and FDR-adjusted q-values, are provided in Supplementary Table S7).

**FIGURE 8:**
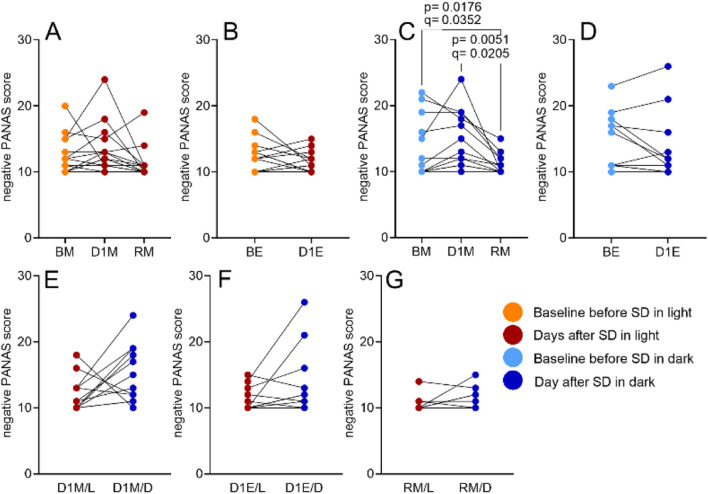
The impact of SD and lighting conditions during SD on the negative PANAS score. Scores were compared for the morning before SD (BM), the morning after SD (D1M), and the morning after recovery sleep (RM; panels **A, C**), as well as for the evening before SD (BE) and the evening after SD (D1E; panels **B, D**), using RM one-way ANOVA. Between-session comparisons of corresponding time points are shown in panels **E–G**. See Materials and Methods for details. p-values refer to the uncorrected results of the RM one-way ANOVA; q-values indicate significance after Benjamini–Hochberg FDR correction.

Negative affect did not change post-SD in SD/L and SD/D groups (F (2.640, 44.87) = 2.334, p = 0.0939, *R* ^2^ = 0.1207; Figures 8A,B,D), but decreased after recovery sleep in the SD/D group (F (2.112, 23.23) = 3.889, p = 0.0330, *R* ^2^ = 0.2612; Figure 8C). Between-session analysis also showed a significant difference (F (2.212, 24.33) = 4.010, p = 0.0279, *R* ^2^ = 0.2672), however, no baseline differences (BM/L vs. BM/D) or group differences (SD/L vs. SD/D on D1) were observed with Benjamini–Hochberg FDR correction (Figures 8E–G; full statistics, including mean differences, 95% CI, uncorrected p-values, and FDR-adjusted q-values, are provided in Supplementary Table S8).

**FIGURE 9:**
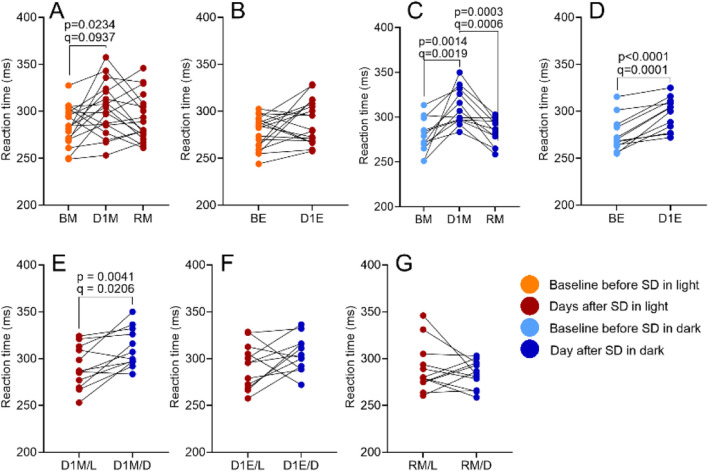
The impact of SD and lighting conditions during SD on the SRT score. Scores were compared for the morning before SD (BM), the morning after SD (D1M), and the morning after recovery sleep (RM; panels **A, C**), as well as for the evening before SD (BE) and the evening after SD (D1E; panels **B, D**). Between-session comparisons of corresponding time points are shown in panels **E–G**. See Materials and Methods for details. p-values refer to the uncorrected results of the RM one-way ANOVA; q-values indicate significance after Benjamini–Hochberg FDR correction.

SRT tests, conducted twice daily, showed significantly longer reaction times in the SD/L group (F (2.580, 43.86) = 4.205, p = 0.0141, *R* ^2^ = 0.1983; Figures 9A,B) and in the SD/D group (F (2,561, 28,18) = 16,51, p < 0.0001, *R* ^2^ = 0.6002; Figures 9C,D). Between-session analysis also showed a significant difference (F (4.229, 46.52) = 5.673, p = 0.0007, *R* ^2^ = 0.3403). No baseline differences were found between BM/L and BM/D or BE/L and BE/D. However, a significant increase in SD/D reaction times, confirmed by Benjamini–Hochberg FDR correction, suggests an effect of SD in darkness (Figures 9E–G; Full statistics, including mean differences, 95% CI, uncorrected p-values, and FDR-adjusted q-values, are provided in Supplementary Table S9).

### 3.3 Effect of lighting regime during SD on glycaemia and hunger sensation

**FIGURE 10:**
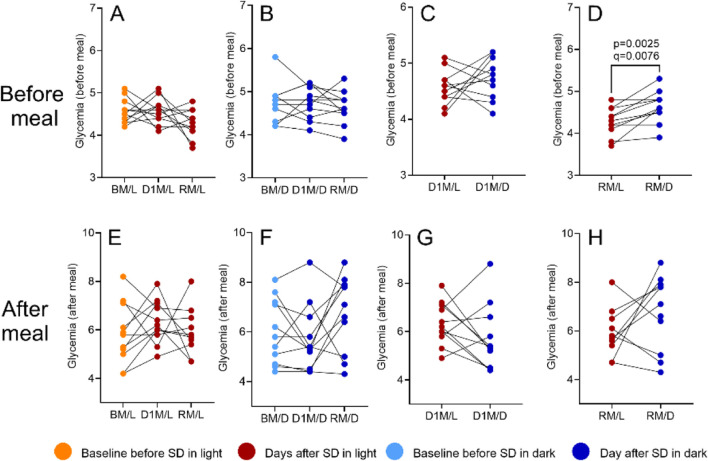
The impact of SD and lighting conditions during SD on the glycaemia. Glycaemia was measured before meal (panels **A–D**) and after meal (panels **E–H**). Values were compared for the morning before SD (BM), the morning after SD (D1M), and the morning after recovery sleep (RM), using RM one-way ANOVA. Between-session comparisons of corresponding time points are shown in panels **C, D** (before meal) and **G, H** (after meal). See Materials and Methods for details. p-values refer to the uncorrected results of the RM one-way ANOVA; q-values indicate significance after Benjamini–Hochberg FDR correction.

Blood glucose levels, measured daily before breakfast, were not significantly affected by SD in either session (RM one-way ANOVA, all p > 0.05; Figures 10A,B). Similarly, no significant changes were found for glycaemia after meal (Figures 10E,F). However, before-meal glucose levels on RM differed significantly (F (3.166, 31.66) = 3.036, p = 0.0411, *R* ^2^ = 0.2329; Figure 10D), suggesting a delayed effect of lighting conditions during SD, as no significant baseline differences between SD/L and SD/D groups were found before meals. (Figures 10C,D,G,H; full *post hoc* results, including mean differences, 95% CI, p- and q-values, are provided in Supplementary Table S10).

Hunger and appetite for specific tastes (sweet, salty, piquant, greasy) were assessed using VAS. Hunger ratings differed significantly from baseline on D1 and RM in both sessions. SD did not affect sweet or salty preferences, though piquant preference decreased post-recovery sleep in the SD/D group. Greasy appetite increased after breakfast on D1 in the SD/D group (Supplementary Table S11).

**FIGURE 11:**
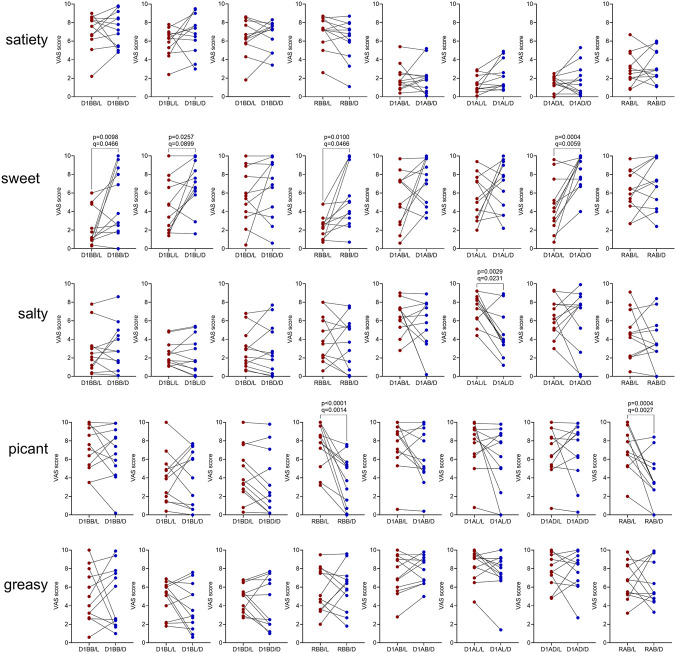
The impact of lighting conditions during SD on hunger and appetite. Scores were compared between SD/L and SD/D groups after SD before breakfast (D1BB, RBB), before lunch (D1BL), before dinner (D1BD), after breakfast (D1AB, RAB), after lunch (D1AL), and after dinner (D1AD). See Materials and Methods for details. p-values refer to the uncorrected results of the RM one-way ANOVA; q-values indicate significance after Benjamini–Hochberg FDR correction.

Baseline comparisons between SD/L and SD/D groups showed no significant differences for hunger or taste preferences, except for salty taste (RM one-way ANOVA: BAD/L vs. BAD/D: p = 0.0077, q = 0.0359). Differences in SD/L and SD/D groups (Figure 11) thus likely reflect lighting conditions during SD. Sweet preference was higher in SD/D, while salty preference was lower after lunch on D1 and piquant preference decreased after recovery sleep. Greasy preference was unaffected by lighting conditions (Full statistics, including RM ANOVA main effects, FDR-adjusted *post hoc* comparisons (Benjamini–Hochberg), and uncorrected Fisher’s LSD tests where appropriate, are provided in Supplementary Table S12).

Exploratory correlations between circadian body temperature rhythm parameters and affected outcomes identified a single FDR-significant result: fasting glycaemia on the recovery day in the SD/L group, which, however, did not correlate with the amplitude of the activity rhythm (Supplementary Figure S1).

## 4 Discussion

This study aimed to compare the immediate and medium-term effects of total SD in illuminated versus dark conditions. Given that aberrant light exposure impairs melatonin production, disrupts circadian regulation and affects brain regions involved in emotion and mood control (Bedrosian and Nelson, 2017), we hypothesized that SD in darkness is less detrimental to circadian regulation and melatonin production. To minimise disruption of natural sleep, melatonin sampling was conducted only on SD nights. As expected, melatonin profiles confirmed that nighttime light significantly suppressed melatonin production compared to dark conditions.

To assess the effects of light or dark during SD on the circadian system, we monitored peripheral temperature 5 days pre- and post-SD. Both conditions impacted these rhythms, but a faster return to baseline was observed in the SD/D group. Comparing the circadian parameters, we observed a notable increase in the mesor on D1 in the SD/D group and on D2 in the SD/L group, along with a transient decrease in amplitude, reflecting higher daytime peripheral temperature minima. Wrist skin temperature, inversely related to core body temperature (CBT), rises during sleep to aid heat loss (Kräuchi and Wirz-Justice, 1994). Evening light slows this rise, while morning light accelerates CBT rise and decreases peripheral temperature (Te Kulve et al., 2016; Lok et al., 2022). Limited data exist on how SD affects skin temperature in humans; however, recent data in young men showed that 24-h SD did not alter whole-body heat exchange or core temperature, indicating preserved central thermoregulation (Koetje et al., 2025). To our knowledge, no delayed effects of nocturnal light exposure or SD on thermoregulation have been observed, except for reports linking increased slow-wave sleep during RS to higher body temperature (Dijk and Czeisler, 1993). Thus our finding may guide further investigation of how nocturnal light modulates peripheral thermoregulatory responses and their circadian dynamics.

Peripheral temperature rhythm acrophase advanced on D1 in the SD/L group, consistent with actigraphic recordings. Previous studies, however, report phase delays in melatonin rhythm during dim-light SD (Cajochen et al., 2003). Elevated serotonin levels during SD, as shown in humans (Davies et al., 2014), may explain the phase advances observed here, given serotonin’s role in circadian clock advancement (Prosser, 2003). Although light exposure throughout the night is not expected to cause a significant phase shift according to the light phase response curve, it might amplify SD effects beyond statistical thresholds. However, the transient phase advance in activity rhythm might have a simpler explanation: reduced activity in the hours before the 10 p.m. bedtime during experiment could be interpreted as a phase advance in sleep timing.

Following SD, decreased activity rhythm mesors reflected a general reduction in activity levels. Amplitude reductions were more pronounced in the SD/L group, aligning with prior findings on light’s additive effect during SD (Dijk et al., 2012). Activity and temperature rhythms, as assessed by the two-way ANOVA, showed deviations from baseline for up to five and 4 days, respectively, in the SD/L group, while the SD/D group normalized by D3/D4. On D5, the activity rhythm again deviated from baseline, likely reflecting changes in the participants’ daily routine, as suggested by distinct activity patterns in the records. These findings show that total SD disrupts circadian rhythms of body temperature and physical activity, with SD in darkness enabling a faster return to pre-SD rhythms. In contrast, circadian rhythms after SD in light remain misaligned with pre-SD parameters even after 5 days.

SD negatively impacts cognitive function and mood regulation (Killgore, 2010; Groeger et al., 2022; Maleček et al., 2023). In our study, SD increased sleepiness and reduced positive affect in both groups on D1, and also prolonged reaction times in the SRT test. Notably, enhanced negative affect in the morning and prolonged evening reaction times on D1 were observed only in the SD/D group. Given that the slopes of most regression lines are comparable to those in the SD/L group, the observed differences may partly reflect the unequal group sizes in this comparison. Overall, these results are consistent with previous reports indicating minimal effects of SD on negative affect (Saksvik-Lehouillier et al., 2020; Stenson et al., 2021), although recent studies report increased negative affect during SD in individuals with late chronotypes (Cox et al., 2024). Light at night, particularly high-CCT or bright light reduces sleepiness, enhances alertness and mood, and improves cognitive performance (Münch et al., 2016; Motamedzadeh et al., 2017; Scheuermaier et al., 2018; Sunde et al., 2022). Consistent with these findings, our results showed lower morning sleepiness and better SRT performance in the SD/L group. Differences in positive and negative affect scores between groups did not reach statistical significance. Although the night-time activities were matched in terms of arousal level, as participants in both SD/L and SD/D were kept awake through conversation or engaging podcasts, we acknowledge that even subtle differences in night-time stimulation may influence next-morning sleepiness and SRT performance.

SD and circadian disruption are linked to metabolic issues and obesity (Benedict et al., 2012; Greer et al., 2013; Liu et al., 2022; McHill et al., 2022; Meléndez-Fernández et al., 2023). In particular, sleep restriction has been associated with a higher likelihood of consuming sweet snacks (Nedeltcheva et al., 2009; Heath et al., 2012), while simulated night shifts increased preference for high-fat breakfast foods (Cain et al., 2015). Our data do not show statistically significant evidence for increased sweet preference after any SD sessions. Spicy preference rose before dinner in the SD/L group and before breakfast on the recovery day in the SD/D group, while fatty food preference rose only after breakfast on D1 in the SD/D group. Nevertheless, our results indicate heightened sweet cravings in the SD/D group compared to SD/L, alongside a milder but decreasing trend in salty after lunch in D1, and spicy cravings, particularly before and after breakfast on the recovery day. Although no direct link between taste preference and nighttime light exposure has been studied, the sweet preference could stem from insulin disturbances, as seen in a study on humans exposed to 100 lux of light at night during sleep (Mason et al., 2022).

Glycaemia was not elevated after SD, consistent with findings that sleep duration alone does not primarily determine glucose metabolism (Kothari et al., 2021; Tsereteli et al., 2022). We did, however, identified a small yet significant difference in preprandial glycaemia levels between the SD/L and SD/D groups after RS. This effect may relate to increased slow-wave sleep (SWS) during RS, which has been associated with altered glucose and insulin dynamics (Scheen et al., 1996; Van Cauter et al., 2008; Ukraintseva et al., 2020). These findings suggest a connection between SWS and glucose metabolism but do not explain the observed differences between SD/L and SD/D groups. One possibility is a rebound effect of melatonin suppression during SD, potentially influencing insulin secretion and next-morning glucose levels (Martorina and Tavares, 2023). If confirmed, this would suggest a delayed metabolic impact of nocturnal light exposure, despite its acute benefits for vigilance and mood.

A limitation of our study is its exclusive focus on male participants, necessitating caution when interpreting the findings due to potential differences in the effects of SD on women, particularly regarding metabolism (Markwald et al., 2013). Additionally, the participant group was relatively homogeneous in age, physical fitness, and lifestyle, which further constrains the generalisability of the findings to broader populations. Another limitation is that we did not assess cognition, sleepiness, or appetite during the SD night. The study focused on next-day effects of nocturnal wakefulness under different lighting conditions, as overnight testing would have disrupted the protocol and was largely unfeasible, especially where maintaining darkness throughout the night was essential. However, a key strength of our design is the homogeneity of the participant group, comprising similarly aged, healthy men with comparable physical fitness and work routines, all consuming a uniform, standardised diet. This approach likely minimises variables that could confound the outcomes of similar studies. Another limitation is the small sample size of 12 participants in the second phase, reflecting the inherent challenges of sleep deprivation research, such as logistical complexity and associated risks, which led to a 30% attrition rate after the first phase. However, the well-controlled repeated-measures design, allowing for paired comparisons, helps mitigate this limitation and supports the reliability of the observed effects despite the smaller sample size.

Insufficient sleep, whether due to restriction or deprivation, is increasingly common in modern society, negatively impacting wellbeing and public health. Our findings suggest that light exposure during SD was associated with lower subjective tiredness, reflected in reduced morning sleepiness, faster SRT reaction times in the morning on D1, and lower sweet cravings, compared to SD in darkness. However, these immediate benefits must be weighed against the potentially harmful effects on the circadian system, as light exposure during SD prolonged the alterations in circadian rhythms. While SD alone temporarily altered diurnal rhythms of peripheral temperature and activity, these changes were more pronounced and lasted longer under nocturnal light. The robustness of these results is supported by converging outcomes across ANOVA, FDR correction, and confidence interval analyses. Given that a stable circadian system is vital for health, our study highlights the trade-off between the short-term benefits and the medium-term risks of nighttime light exposure.

## Data Availability

The original contributions presented in the study are included in the article/Supplementary Material, further inquiries can be directed to the corresponding author.

## References

[B1] Albreiki M. S. Shamlan G. H. BaHammam A. S. Alruwaili N. W. Middleton B. Hampton S. M. (2022). Acute impact of light at night and exogenous melatonin on subjective appetite and plasma leptin. Front. Nutr. 9, 1079453. 10.3389/fnut.2022.1079453 36562040 PMC9763572

[B2] Amaral F. Cipolla-Neto J. (2018). A brief review about melatonin, a pineal hormone. Arch. Endocrinol. Metab. 62, 472–479. 10.20945/2359-3997000000066 30304113 PMC10118741

[B3] Bedrosian T. A. Nelson R. J. (2017). Timing of light exposure affects mood and brain circuits. Transl. Psychiatry 7, e1017. 10.1038/tp.2016.262 28140399 PMC5299389

[B4] Benedict C. Brooks S. J. O’Daly O. G. Almèn M. S. Morell A. Åberg K. (2012). Acute sleep deprivation enhances the brain’s response to Hedonic food stimuli: an fMRI study. J. Clin. Endocrinol. Metab. 97, E443–E447. 10.1210/jc.2011-2759 22259064

[B5] Cain S. W. Filtness A. J. Phillips C. L. Anderson C. (2015). Enhanced preference for high-fat foods following a simulated night shift. Scand. J. Work, Environ. and Health 41, 288–293. 10.5271/sjweh.3486 25699635

[B6] Cain S. W. McGlashan E. M. Vidafar P. Mustafovska J. Curran S. P. N. Wang X. (2020). Evening home lighting adversely impacts the circadian system and sleep. Sci. Rep. 10, 19110. 10.1038/s41598-020-75622-4 33154450 PMC7644684

[B7] Cajochen C. Jewett M. E. Dijk D.-J. (2003). Human circadian melatonin rhythm phase delay during a fixed sleep–wake schedule interspersed with nights of sleep deprivation. J. Pineal Res. 35, 149–157. 10.1034/j.1600-079X.2003.00072.x 12932197

[B8] Cajochen C. Stephani O. Schollhorn I. Lang D. Chellappa S. L. (2022). Influence of evening light exposure on polysomnographically assessed night-time sleep: a systematic review with meta-analysis. Light. Res. Technol. 54, 513–624. 10.1177/14771535221078765

[B9] Conkright W. R. Beckner M. E. Sinnott A. M. Eagle S. R. Martin B. J. Lagoy A. D. (2021). Neuromuscular performance and hormonal responses to military operational stress in men and women. J. Strength Cond. Res. 35, 1296–1305. 10.1519/JSC.0000000000004013 33780395

[B10] Cox R. C. Ritchie H. K. Knauer O. A. Guerin M. K. Stothard E. R. Wright K. P. (2024). Chronotype and affective response to sleep restriction and subsequent sleep deprivation. J. Biol. Rhythms 39, 35–48. 10.1177/07487304231188204 37539684 PMC10838359

[B11] Davies S. K. Ang J. E. Revell V. L. Holmes B. Mann A. Robertson F. P. (2014). Effect of sleep deprivation on the human metabolome. Proc. Natl. Acad. Sci. U. S. A. 111, 10761–10766. 10.1073/pnas.1402663111 25002497 PMC4115565

[B12] Dijk D. J. Czeisler C. A. (1993). Body temperature is elevated during the rebound of slow-wave sleep following 40-h of sleep deprivation on a constant routine. J. Sleep. Res. 2, 117–120. 10.1111/j.1365-2869.1993.tb00073.x 10607081

[B13] Dijk D.-J. Duffy J. F. Silva E. J. Shanahan T. L. Boivin D. B. Czeisler C. A. (2012). Amplitude reduction and phase shifts of melatonin, cortisol and other circadian rhythms after a gradual advance of sleep and light exposure in humans. PLoS ONE 7, e30037. 10.1371/journal.pone.0030037 22363414 PMC3281823

[B14] Flint A. Raben A. Blundell J. E. Astrup A. (2000). Reproducibility, power and validity of visual analogue scales in assessment of appetite sensations in single test meal studies. Int. J. Obes. Relat. Metab. Disord. 24, 38–48. 10.1038/sj.ijo.0801083 10702749

[B15] Gooley J. J. Chamberlain K. Smith K. A. Khalsa S. B. S. Rajaratnam S. M. W. Van Reen E. (2011). Exposure to room light before bedtime suppresses melatonin onset and shortens melatonin duration in humans. J. Clin. Endocrinol. Metab. 96, E463–E472. 10.1210/jc.2010-2098 21193540 PMC3047226

[B16] Greer S. M. Goldstein A. N. Walker M. P. (2013). The impact of sleep deprivation on food desire in the human brain. Nat. Commun. 4, 2259. 10.1038/ncomms3259 23922121 PMC3763921

[B17] Groeger J. A. Lo J. C.-Y. Santhi N. Lazar A. S. Dijk D.-J. (2022). Contrasting effects of sleep restriction, total sleep deprivation, and sleep timing on positive and negative affect. Front. Behav. Neurosci. 16, 911994. 10.3389/fnbeh.2022.911994 36062257 PMC9433122

[B18] Gumenyuk V. Roth T. Drake C. L. (2012). Circadian phase, sleepiness, and light exposure assessment in night workers with and without shift work disorder. Chronobiol Int. 29, 928–936. 10.3109/07420528.2012.699356 22823876

[B19] Heath G. Roach G. D. Dorrian J. Ferguson S. A. Darwent D. Sargent C. (2012). The effect of sleep restriction on snacking behaviour during a week of simulated shiftwork. Accid. Analysis and Prev. 45, 62–67. 10.1016/j.aap.2011.09.028 22239934

[B20] Hoddes E. Zarcone V. Smythe H. Phillips R. Dement W. C. (1973). Quantification of sleepiness: a new approach. Psychophysiology 10, 431–436. 10.1111/j.1469-8986.1973.tb00801.x 4719486

[B21] Horne J. A. Ostberg O. (1976). A self-assessment questionnaire to determine morningness-eveningness in human circadian rhythms. Int. J. Chronobiol 4, 97–110. Available online at: https://pubmed.ncbi.nlm.nih.gov/1027738/. 1027738

[B22] Killgore W. D. S. (2010). Effects of sleep deprivation on cognition. Prog. Brain Res. 185, 105–129. 10.1016/B978-0-444-53702-7.00007-5 21075236

[B23] Koetje N. J. Kirby N. V. O’Connor F. K. Richards B. J. Janetos K.-M. T. Ioannou L. G. (2025). Effects of 24-h sleep deprivation on whole-body heat exchange in young men during exercise in the heat. Eur. J. Appl. Physiol. 125, 1565–1576. 10.1007/s00421-025-05705-5 39875706

[B24] Kothari V. Cardona Z. Chirakalwasan N. Anothaisintawee T. Reutrakul S. (2021). Sleep interventions and glucose metabolism: systematic review and meta-analysis. Sleep. Med. 78, 24–35. 10.1016/j.sleep.2020.11.035 33383394

[B25] Kräuchi K. (2007). The thermophysiological Cascade leading to sleep initiation in relation to phase of entrainment. Sleep. Med. Rev. 11, 439–451. 10.1016/j.smrv.2007.07.001 17764994

[B26] Kräuchi K. Wirz-Justice A. (1994). Circadian rhythm of heat production, heart rate, and skin and core temperature under unmasking conditions in men. Am. J. Physiol. 267, R819–R829. 10.1152/ajpregu.1994.267.3.R819 8092328

[B27] Liew S. C. Aung T. (2021). Sleep deprivation and its association with diseases-a review. Sleep. Med. 77, 192–204. 10.1016/j.sleep.2020.07.048 32951993

[B28] Liu S. Wang X. Zheng Q. Gao L. Sun Q. (2022). Sleep deprivation and central appetite regulation. Nutrients 14, 5196. 10.3390/nu14245196 36558355 PMC9783730

[B29] Lok R. Woelders T. van Koningsveld M. J. Oberman K. Fuhler S. G. Beersma D. G. M. (2022). Bright light decreases peripheral skin temperature in healthy men: a forced desynchrony study under dim and bright light (II). J. Biol. Rhythms 37, 417–428. 10.1177/07487304221096948 35723003 PMC9326805

[B30] Maleček J. Omcirk D. Skálová K. Pádecký J. Janikov M. T. Obrtel M. (2023). Effects of 36 hours of sleep deprivation on military-related tasks: can ammonium inhalants maintain performance? PLoS One 18, e0293804. 10.1371/journal.pone.0293804 37967128 PMC10651003

[B31] Markwald R. R. Melanson E. L. Smith M. R. Higgins J. Perreault L. Eckel R. H. (2013). Impact of insufficient sleep on total daily energy expenditure, food intake, and weight gain, Proc. Natl. Acad. Sci. U. S. A., 110, 5695–5700. 10.1073/pnas.1216951110 23479616 PMC3619301

[B32] Martorina W. Tavares A. (2023). Glycemic variability in patients with type 2 diabetes mellitus (T2DM): the role of melatonin in a crossover, double-blind, placebo-controlled, randomized study. Nutrients 15, 3523. 10.3390/nu15163523 37630714 PMC10458393

[B33] Mason I. C. Grimaldi D. Reid K. J. Warlick C. D. Malkani R. G. Abbott S. M. (2022). Light exposure during sleep impairs cardiometabolic function. Proc. Natl. Acad. Sci. 119, e2113290119. 10.1073/pnas.2113290119 35286195 PMC8944904

[B34] McHill A. W. Hull J. T. Klerman E. B. (2022). Chronic circadian disruption and sleep restriction influence subjective hunger, appetite, and food preference. Nutrients 14, 1800. 10.3390/nu14091800 35565768 PMC9105437

[B35] Meléndez-Fernández O. H. Liu J. A. Nelson R. J. (2023). Circadian rhythms disrupted by light at night and mistimed food intake alter hormonal rhythms and metabolism. Int. J. Mol. Sci. 24, 3392. 10.3390/ijms24043392 36834801 PMC9963929

[B36] Motamedzadeh M. Golmohammadi R. Kazemi R. Heidarimoghadam R. (2017). The effect of blue-enriched white light on cognitive performances and sleepiness of night-shift workers: a field study. Physiol. Behav. 177, 208–214. 10.1016/j.physbeh.2017.05.008 28495465

[B37] Münch M. Nowozin C. Regente J. Bes F. De Zeeuw J. Hädel S. (2016). Blue-enriched morning light as a countermeasure to light at the wrong time: effects on cognition, sleepiness, sleep, and circadian phase. Neuropsychobiology 74, 207–218. 10.1159/000477093 28637029

[B38] Nedeltcheva A. V. Kilkus J. M. Imperial J. Kasza K. Schoeller D. A. Penev P. D. (2009). Sleep curtailment is accompanied by increased intake of calories from snacks. Am. J. Clin. Nutr. 89, 126–133. 10.3945/ajcn.2008.26574 19056602 PMC2615460

[B39] Phillips A. J. K. Vidafar P. Burns A. C. McGlashan E. M. Anderson C. Rajaratnam S. M. W. (2019). High sensitivity and interindividual variability in the response of the human circadian system to evening light. Proc. Natl. Acad. Sci. 116, 12019–12024. 10.1073/pnas.1901824116 31138694 PMC6575863

[B40] Prosser R. A. (2003). Serotonin phase-shifts the mouse suprachiasmatic circadian clock *in vitro* . Brain Res. 966, 110–115. 10.1016/s0006-8993(02)04206-3 12646314

[B41] Saksvik-Lehouillier I. Saksvik S. B. Dahlberg J. Tanum T. K. Ringen H. Karlsen H. R. (2020). Mild to moderate partial sleep deprivation is associated with increased impulsivity and decreased positive affect in young adults. Sleep 43, zsaa078. 10.1093/sleep/zsaa078 32306048 PMC7551297

[B42] Scheen A. J. Byrne M. M. Plat L. Leproult R. Van Cauter E. (1996). Relationships between sleep quality and glucose regulation in normal humans. Am. J. Physiol. 271, E261–E270. 10.1152/ajpendo.1996.271.2.E261 8770019

[B43] Scheer F. A. J. L. Hilton M. F. Mantzoros C. S. Shea S. A. (2009). Adverse metabolic and cardiovascular consequences of circadian misalignment. Proc. Natl. Acad. Sci. U. S. A. 106, 4453–4458. 10.1073/pnas.0808180106 19255424 PMC2657421

[B44] Scheuermaier K. Münch M. Ronda J. M. Duffy J. F. (2018). Improved cognitive morning performance in healthy older adults following blue-enriched light exposure on the previous evening. Behav. Brain Res. 348, 267–275. 10.1016/j.bbr.2018.04.021 29684473 PMC6124504

[B45] Šmotek M. Fárková E. Manková D. Kopřivová J. (2020). Evening and night exposure to screens of media devices and its association with subjectively perceived sleep: should “light hygiene” be given more attention? Sleep. Health 6, 498–505. 10.1016/j.sleh.2019.11.007 32197951

[B46] Stenson A. R. Kurinec C. A. Hinson J. M. Whitney P. Van Dongen H. P. A. (2021). Total sleep deprivation reduces top-down regulation of emotion without altering bottom-up affective processing. PLoS One 16, e0256983. 10.1371/journal.pone.0256983 34473768 PMC8412406

[B47] Sunde E. Mrdalj J. Pedersen T. T. Bjorvatn B. Grønli J. Harris A. (2022). Bright light exposure during simulated night work improves cognitive flexibility. Chronobiol Int. 39, 948–963. 10.1080/07420528.2022.2050922 35343353

[B48] Te Kulve M. Schellen L. Schlangen L. J. M. van Marken Lichtenbelt W. D. (2016). The influence of light on thermal responses. Acta Physiol. (Oxf) 216, 163–185. 10.1111/apha.12552 26172218

[B49] Tsereteli N. Vallat R. Fernandez-Tajes J. Delahanty L. M. Ordovas J. M. Drew D. A. (2022). Impact of insufficient sleep on dysregulated blood glucose control under standardised meal conditions. Diabetologia 65, 356–365. 10.1007/s00125-021-05608-y 34845532 PMC8741723

[B50] Ukraintseva Yu. V. Liaukovich K. M. Saltykov K. A. Belov D. A. Nizhnik А. N. (2020). Selective slow-wave sleep suppression affects glucose tolerance and melatonin secretion. The role of sleep architecture. Sleep. Med. 67, 171–183. 10.1016/j.sleep.2019.11.1254 31935619

[B51] Vallat R. Berry S. E. Tsereteli N. Capdevila J. Khatib H. A. Valdes A. M. (2022). How people wake up is associated with previous night’s sleep together with physical activity and food intake. Nat. Commun. 13, 7116. 10.1038/s41467-022-34503-2 36402781 PMC9675783

[B52] Van Cauter E. Spiegel K. Tasali E. Leproult R. (2008). Metabolic consequences of sleep and sleep loss. Sleep. Med. 9 (Suppl. 1), S23–S28. 10.1016/S1389-9457(08)70013-3 18929315 PMC4444051

[B53] van Marken Lichtenbelt W. D. Daanen H. A. M. Wouters L. Fronczek R. Raymann R. J. E. M. Severens N. M. W. (2006). Evaluation of wireless determination of skin temperature using iButtons. Physiol. Behav. 88, 489–497. 10.1016/j.physbeh.2006.04.026 16797616

[B54] Walker W. H. Walton J. C. DeVries A. C. Nelson R. J. (2020). Circadian rhythm disruption and mental health. Transl. Psychiatry 10, 28. 10.1038/s41398-020-0694-0 32066704 PMC7026420

[B55] Watson D. Clark L. A. Tellegen A. (1988). Development and validation of brief measures of positive and negative affect: the PANAS scales. J. Pers. Soc. Psychol. 54, 1063–1070. 10.1037//0022-3514.54.6.1063 3397865

