# Supplementary material for: The effects of total sleep deprivation on the circadian rhythms and psychophysiological factors in military cadets; a comparison between wakefulness in light and darkness
Source: Front Physiol. 2026 Jan 16;16:1732257. doi: 10.3389/fphys.2025.1732257 (PMC12855117; doi:10.3389/fphys.2025.1732257)
Supplement: Supplementary file 1 [file Table1.docx]

**Supplementary Information for**: **The effects of total sleep deprivation on the circadian rhythms and psychophysiological factors in military cadets; a comparison between wakefulness in light and darkness**

Kateřina Skálová^1.2^, Jan Maleček^3^, David Kolář^1^, Kateřina Červená^1^, Jana Kopřivová^1.4^, James Tufano^3^, Dan Omcirk^3^, Jan Padecký^3^, Tomas Vetrovsky^3^, Zdeňka Bendová^1.2.4*^

^1^ National Institute of Mental Health, Klecany, Czech Republic.

^2^ Faculty of Science, Charles University, Prague, Czech Republic

^3^ Faculty of Physical Education and Sport, Charles University, Prague, Czech Republic

^4^ Third Faculty of Medicine, Charles University, Prague. Czech Republic

**Table 1. Statistics for wrist skin temperature measurements.** One-way RM ANOVA comparing groups within the SD/L and SD/D conditions, with Benjamini–Hochberg FDR correction and uncorrected Fisher’s LSD applied where appropriate.

| Number of families | 1 |  |  |  |  |  |  |  |
| --- | --- | --- | --- | --- | --- | --- | --- | --- |
| Number of comparisons per family | 5 |  |  |  |  |  |  |  |
| Q | 0.05 |  |  |  |  |  |  |  |
|  |  |  |  |  |  |  |  |  |
| **Original FDR method of  Benjamini and Hochberg** | Mean diff, | Discovery? | q value | Individual P Value | A-? |  |  |  |
| SD/L, BD vs. SD/L D1 | 0.1713 | **Yes** | **0.0136** | **0.0109** | B | SD/L D1 |  |  |
| SD/L, BD vs. SD/L D2 | -0.1278 | **Yes** | **0.0020** | **0.0012** | C | SD/L D2 |  |  |
| SD/L, BD vs. SD/L D3 | 0.1919 | **Yes** | **<0.0001** | **<0.0001** | D | SD/L D3 |  |  |
| SD/L, BD vs. SD/L D4 | 0.2639 | **Yes** | **<0.0001** | **<0.0001** | E | SD/L D4 |  |  |
| SD/L, BD vs. SD/L D5 | -0.07818 | **Yes** | **0.0304** | **0.0304** | F | SD/L D5 |  |  |
|  |  |  |  |  |  |  |  |  |
| Test details | Mean 1 | Mean 2 | Mean diff, | SE of diff, | n1 | n2 | t | DF |
| SD/L, BD vs. SD/L D1 | 34.06 | 33.89 | 0.1713 | 0.06591 | 96 | 96 | 2.599 | 95 |
| SD/L, BD vs. SD/L D2 | 34.06 | 34.19 | -0.1278 | 0.03822 | 96 | 96 | 3.345 | 95 |
| SD/L, BD vs. SD/L D3 | 34.06 | 33.87 | 0.1919 | 0.03173 | 96 | 96 | 6.046 | 95 |
| SD/L, BD vs. SD/L D4 | 34.06 | 33.79 | 0.2639 | 0.02986 | 96 | 96 | 8.838 | 95 |
| SD/L, BD vs. SD/L D5 | 34.06 | 34.14 | -0.07818 | 0.03557 | 96 | 96 | 2.198 | 95 |

| Number of families | 1 |  |  |  |  |  |  |  |
| --- | --- | --- | --- | --- | --- | --- | --- | --- |
| Number of comparisons per family | 5 |  |  |  |  |  |  |  |
| Alpha | 0.05 |  |  |  |  |  |  |  |
|  |  |  |  |  |  |  |  |  |
| **Uncorrected Fisher's LSD** | Mean diff, | 95.00% CI of diff, | Below threshold? | Summary | Individual P Value | A-? |  |  |
| SD/L, BD vs. SD/L D1 | 0.1713 | 0.04042 to 0.3021 | Yes | * | 0.0109 | B | SD/L D1 |  |
| SD/L, BD vs. SD/L D2 | -0.1278 | -0.2037 to -0.05195 | Yes | ** | 0.0012 | C | SD/L D2 |  |
| SD/L, BD vs. SD/L D3 | 0.1919 | 0.1289 to 0.2549 | Yes | **** | <0.0001 | D | SD/L D3 |  |
| SD/L, BD vs. SD/L D4 | 0.2639 | 0.2046 to 0.3231 | Yes | **** | <0.0001 | E | SD/L D4 |  |
| SD/L, BD vs. SD/L D5 | -0.07818 | -0.1488 to -0.007559 | Yes | * | 0.0304 | F | SD/L D5 |  |
|  |  |  |  |  |  |  |  |  |

| Number of families | 1 |  |  |  |  |  |  |  |
| --- | --- | --- | --- | --- | --- | --- | --- | --- |
| Number of comparisons per family | 5 |  |  |  |  |  |  |  |
| Q | 0.05 |  |  |  |  |  |  |  |
|  |  |  |  |  |  |  |  |  |
| **Original FDR method of**  **Benjamini and Hochberg** | Mean diff, | Discovery? | q value | Individual P Value | A-? |  |  |  |
| SD/D, BD vs. SD/D, D1 | -0.2811 | **Yes** | **<0.0001** | **<0.0001** | B | SD/D, D1 |  |  |
| SD/D, BD vs. SD/D, D2 | -0.1443 | **Yes** | **0.0322** | **0.0129** | C | SD/D, D2 |  |  |
| SD/D, BD vs. SD/D, D3 | 0.05862 | No | 0.3884 | 0.3107 | D | SD/D, D3 |  |  |
| SD/D, BD vs. SD/D, D4 | -0.04204 | No | 0.4671 | 0.4671 | E | SD/D, D4 |  |  |
| SD/D, BD vs. SD/D, D5 | 0.06427 | No | 0.3884 | 0.2665 | F | SD/D, D5 |  |  |
|  |  |  |  |  |  |  |  |  |
| Test details | Mean 1 | Mean 2 | Mean diff, | SE of diff, | n1 | n2 | t | DF |
| SD/D, BD vs. SD/D, D1 | 34.07 | 34.36 | -0.2811 | 0.05777 | 95 | 95 | 4.865 | 470 |
| SD/D, BD vs. SD/D, D2 | 34.07 | 34.22 | -0.1443 | 0.05777 | 95 | 95 | 2.497 | 470 |
| SD/D, BD vs. SD/D, D3 | 34.07 | 34.02 | 0.05862 | 0.05777 | 95 | 95 | 1.015 | 470 |
| SD/D, BD vs. SD/D, D4 | 34.07 | 34.12 | -0.04204 | 0.05777 | 95 | 95 | 0.7278 | 470 |
| SD/D, BD vs. SD/D, D5 | 34.07 | 34.01 | 0.06427 | 0.05777 | 95 | 95 | 1.113 | 470 |

| Number of families | 1 |  |  |  |  |  |  |  |
| --- | --- | --- | --- | --- | --- | --- | --- | --- |
| Number of comparisons per family | 5 |  |  |  |  |  |  |  |
| Alpha | 0.05 |  |  |  |  |  |  |  |
|  |  |  |  |  |  |  |  |  |
| **Uncorrected Fisher's LSD** | Mean diff, | 95.00% CI of diff, | Below threshold? | Summary | Individual P Value | A-? |  |  |
| SD/D, BD vs. SD/D, D1 | -0.2811 | -0.3946 to -0.1676 | Yes | **** | <0.0001 | B | SD/D, D1 |  |
| SD/D, BD vs. SD/D, D2 | -0.1443 | -0.2578 to -0.03074 | Yes | * | 0.0129 | C | SD/D, D2 |  |
| SD/D, BD vs. SD/D, D3 | 0.05862 | -0.05490 to 0.1721 | No | ns | 0.3107 | D | SD/D, D3 |  |
| SD/D, BD vs. SD/D, D4 | -0.04204 | -0.1556 to 0.07147 | No | ns | 0.4671 | E | SD/D, D4 |  |
| SD/D, BD vs. SD/D, D5 | 0.06427 | -0.04925 to 0.1778 | No | ns | 0.2665 | F | SD/D, D5 |  |
|  |  |  |  |  |  |  |  |  |

**Table 2: One-way ANOVA statistic with multiple comparisons was used to compare the circadian parameters of the rhythms in wrist skin temperature between baseline and days after SD.**

| Temperature | Tukey´s multiple comparisons test | | | |
| --- | --- | --- | --- | --- |
|  | Mean Diff. | 95.00% CI of diff. | Summary | P value |
| Mesor SD/L | | | | |
| BD vs D1 | 0.07000 | -0.06548 to 0.2055 | ns | 0.6608 |
| BD vs D2 | -0.1200 | -0.2555 to 0.01548 | ns | 0.1128 |
| BD vs D3 | 0.1900 | 0.03553 to 0.3445 | ** | 0.0072 |
| BD vs D4 | 0.2700 | 0.1345 to 0.4055 | **** | <0.0001 |
| BD vs D5 | -0.06000 | -0.1955 to 0.07548 | ns | 0.7888 |
| Mesor SD/D | | | | |
| BD vs D1 | -0.2900 | -0.4619 to -0.1181 | **** | <0.0001 |
| BD vs D2 | -0.1400 | -0.3119 to 0.03194 | ns | 0.1726 |
| BD vs D3 | 0.04000 | -0.1319 to 0.2119 | ns | 0.9826 |
| BD vs D4 | -0.03000 | -0.2019 to 0.1419 | ns | 0.9953 |
| BD vs D5 | 0.05000 | -0.1219 to 0.2219 | ns | 0.9545 |
| Amplitude SD/L | | | | |
| BD vs D1 | 0.2027 | 0.01286 to 0.3925 | * | 0.0293 |
| BD vs D2 | 0.2058 | 0.01596 to 0.3956 | * | 0.0256 |
| BD vs D3 | 0.01430 | -0.1755 to 0.2041 | ns | >0.9999 |
| BD vs D4 | -0.1533 | -0.3431 to 0.03654 | ns | 0.1848 |
| BD vs D5 | 0.1419 | -0.04794 to 0.3317 | ns | 0.2589 |
| Amplitude SD/D | | | | |
| BD vs D1 | 0.09790 | -0.1429 to 0.3387 | ns | 0.8343 |
| BD vs D2 | 0.3774 | 0.1366 to 0.6182 | *** | 0.0003 |
| BD vs D3 | -0.1851 | -0.4259 to 0.05569 | ns | 0.2237 |
| BD vs D4 | -0.07110 | -0.3119 to 0.1697 | ns | 0.9515 |
| BD vs D5 | -0.2091 | -0.4499 to 0.03169 | ns | 0.1237 |
| Acrophase SD/L | | | | |
| BD vs D1 | 0.9000 | 0.04727 to 1.753 | * | 0.0324 |
| BD vs D2 | -0.4300 | -1.283 to 0.4227 | ns | 0.6850 |
| BD vs D3 | 0.08000 | -0.7727 to 0.9327 | ns | 0.9998 |
| BD vs D4 | 0.03000 | -0.8227 to 0.8827 | ns | >0.9999 |
| BD vs D5 | 0.4300 | -0.4227 to 1.283 | ns | 0.6850 |
| Acrophase SD/D | | | | |
| BD vs D1 | 0.9900 | -0.1217 to 2.102 | ns | 0.1072 |
| BD vs D2 | -1.050 | -2.162 to 0.06167 | ns | 0.0744 |
| BD vs D3 | 0.4800 | -0.6317 to 1.592 | ns | 0.7968 |
| BD vs D4 | 0.5200 | -0.5917 to 1.632 | ns | 0.7374 |
| BD vs D5 | -0.8300 | -1.942 to 0.2817 | ns | 0.2521 |

**Table 3: Statistics for activity measurements.** One-way RM ANOVA comparing groups within the SD/L and SD/D conditions, with Benjamini–Hochberg FDR correction and uncorrected Fisher’s LSD applied where appropriate.

| Number of families | 1 |  |  |  |  |  |  |  |
| --- | --- | --- | --- | --- | --- | --- | --- | --- |
| Number of comparisons per family | 5 |  |  |  |  |  |  |  |
| Q | 0.05 |  |  |  |  |  |  |  |
|  |  |  |  |  |  |  |  |  |
| **Original FDR method**  **of Benjamini and Hochberg** | Mean Diff, | Discovery? | q value | Individual P Value | A-? |  |  |  |
| SD/L, BD vs. SD/L D1 | 3004 | **Yes** | **<0.0001** | **<0.0001** | B | SD/L D1 |  |  |
| SD/L, BD vs. SD/L D2 | 1737 | **Yes** | **0.0068** | **0.0041** | C | SD/L D2 |  |  |
| SD/L, BD vs. SD/L D3 | 1439 | **Yes** | **0.0213** | **0.0170** | D | SD/L D3 |  |  |
| SD/L, BD vs. SD/L D4 | 1315 | **Yes** | **0.0290** | **0.0290** | E | SD/L D4 |  |  |
| SD/L, BD vs. SD/L D5 | 1944 | **Yes** | **0.0033** | **0.0013** | F | SD/L D5 |  |  |
|  |  |  |  |  |  |  |  |  |
| Test details | Mean 1 | Mean 2 | Mean Diff, | SE of diff, | n1 | n2 | t | DF |
| SD/L, BD vs. SD/L D1 | 10013 | 7008 | 3004 | 598.6 | 48 | 48 | 5.019 | 235 |
| SD/L, BD vs. SD/L D2 | 10013 | 8276 | 1737 | 598.6 | 48 | 48 | 2.902 | 235 |
| SD/L, BD vs. SD/L D3 | 10013 | 8574 | 1439 | 598.6 | 48 | 48 | 2.403 | 235 |
| SD/L, BD vs. SD/L D4 | 10013 | 8698 | 1315 | 598.6 | 48 | 48 | 2.196 | 235 |
| SD/L, BD vs. SD/L D5 | 10013 | 8068 | 1944 | 598.6 | 48 | 48 | 3.248 | 235 |

| Number of families | 1 |  | |  |  |  | | |  |  |  |
| --- | --- | --- | --- | --- | --- | --- | --- | --- | --- | --- | --- |
| Number of comparisons per family | 5 |  | |  |  |  | | |  |  |  |
| Alpha | 0.05 |  | |  |  |  | | |  |  |  |
|  |  |  | |  |  |  | | |  |  |  |
| **Uncorrected Fisher's LSD** | Mean diff, | 95.00% CI of diff, | | Below threshold? | Summary | Individual P Value | | | A-? |  |  |
| SD/L, BD vs. SD/L D1 | 3004 | 1825 to 4184 | | Yes | **** | <0.0001 | | | B | SD/L D1 |  |
| SD/L, BD vs. SD/L D2 | 1737 | 557.6 to 2916 | | Yes | ** | 0.0041 | | | C | SD/L D2 |  |
| SD/L, BD vs. SD/L D3 | 1439 | 259.3 to 2618 | | Yes | * | 0.0170 | | | D | SD/L D3 |  |
| SD/L, BD vs. SD/L D4 | 1315 | 135.4 to 2494 | | Yes | * | 0.0290 | | | E | SD/L D4 |  |
| SD/L, BD vs. SD/L D5 | 1944 | 765.1 to 3124 | | Yes | ** | 0.0013 | | | F | SD/L D5 |  |
|  |  |  | |  |  |  | | |  |  |  |
|  |  |  | |  |  |  | | |  |  |  |
| Number of families | 1 |  |  |  |  |  |  |  |  |  |  |
| Number of comparisons per family | 5 |  |  |  |  |  |  |  |  |  |  |
| Q | 0.05 |  |  |  |  |  |  |  |  |  |  |
|  |  |  |  |  |  |  |  |  |  |  |  |
| **Original FDR method**  **of Benjamini and Hochberg** | Mean Diff | Discovery? | q value | Individual P Value | A-? |  |  |  |  |  |  |
| SD/D, BD vs. SD/D, D1 | 3499 | **Yes** | **<0.0001** | **<0.0001** | B | SD/D, D1 |  |  |  |  |  |
| SD/D, BD vs. SD/D, D2 | 3566 | **Yes** | **<0.0001** | **<0.0001** | C | SD/D, D2 |  |  |  |  |  |
| SD/D, BD vs. SD/D, D3 | 932.2 | No | 0.0733 | 0.0586 | D | SD/D, D3 |  |  |  |  |  |
| SD/D, BD vs. SD/D, D4 | 800.9 | No | 0.1730 | 0.1730 | E | SD/D, D4 |  |  |  |  |  |
| SD/D, BD vs. SD/D, D5 | 2537 | Yes | 0.0006 | 0.0004 | F | SD/D, D5 |  |  |  |  |  |
|  |  |  |  |  |  |  |  |  |  |  |  |
| Test details | Mean 1 | Mean 2 | Mean Diff, | SE of diff, | n1 | n2 | t | DF |  |  |  |
| SD/D, BD vs. SD/D, D1 | 10289 | 6790 | 3499 | 623.8 | 48 | 48 | 5.609 | 47 |  |  |  |
| SD/D, BD vs. SD/D, D2 | 10289 | 6723 | 3566 | 474.2 | 48 | 48 | 7.520 | 47 |  |  |  |
| SD/D, BD vs. SD/D, D3 | 10289 | 9357 | 932.2 | 481.0 | 48 | 48 | 1.938 | 47 |  |  |  |
| SD/D, BD vs. SD/D, D4 | 10289 | 9488 | 800.9 | 578.9 | 48 | 48 | 1.383 | 47 |  |  |  |
| SD/D, BD vs. SD/D, D5 | 10289 | 7752 | 2537 | 662.7 | 48 | 48 | 3.828 | 47 |  |  |  |

| Number of families | 1 |  |  |  |  |  |  |  |
| --- | --- | --- | --- | --- | --- | --- | --- | --- |
| Number of comparisons per family | 5 |  |  |  |  |  |  |  |
| Alpha | 0.05 |  |  |  |  |  |  |  |
|  |  |  |  |  |  |  |  |  |
| **Uncorrected Fisher's LSD** | Mean diff, | 95.00% CI of diff, | Below threshold? | Summary | Individual P Value | A-? |  |  |
| SD/D, BD vs. SD/D, D1 | 3499 | 2244 to 4754 | Yes | **** | <0.0001 | B | SD/D, D1 |  |
| SD/D, BD vs. SD/D, D2 | 3566 | 2612 to 4520 | Yes | **** | <0.0001 | C | SD/D, D2 |  |
| SD/D, BD vs. SD/D, D3 | 932.2 | -35.37 to 1900 | No | ns | 0.0586 | D | SD/D, D3 |  |
| SD/D, BD vs. SD/D, D4 | 800.9 | -363.7 to 1966 | No | ns | 0.1730 | E | SD/D, D4 |  |
| SD/D, BD vs. SD/D, D5 | 2537 | 1204 to 3870 | Yes | *** | 0.0004 | F | SD/D, D5 |  |
|  |  |  |  |  |  |  |  |  |

**Table 4: One-way ANOVA statistic with multiple comparisons was used to compare the circadian parameters of the rhythms in activity between baseline and days after SD.**

| Actigraphy | Tukey´s multiple comparisons test | | | |
| --- | --- | --- | --- | --- |
|  | Mean Diff. | 95.00% CI of diff. | Summary | P value |
| Mesor SD/L | | | | |
| BD vs D1 | 2880 | 961.1 to 4799 | *** | 0.0005 |
| BD vs D2 | 1689 | -229.9 to 3608 | ns | 0.1173 |
| BD vs D3 | 1563 | -355.9 to 3482 | ns | 0.1773 |
| BD vs D4 | 1158 | -760.9 to 3077 | ns | 0.4982 |
| BD vs D5 | 2026 | 107.1 to 3945 | * | 0.0323 |
| Mesor SD/D | | | | |
| BD vs D1 | 3769 | 1564 to 5974 | **** | <0.0001 |
| BD vs D2 | 3254 | 1049 to 5459 | *** | 0.0007 |
| BD vs D3 | 1111 | -1094 to 3316 | ns | 0.6786 |
| BD vs D4 | 494.0 | -1711 to 2699 | ns | 0.9859 |
| BD vs D5 | 2354 | 149.2 to 4559 | * | 0.0296 |
| Amplitude SD/L | | | | |
| BD vs D1 | 0.2027 | 0.01286 to 0.3925 | * | 0.0293 |
| BD vs D2 | 0.2058 | 0.01596 to 0.3956 | * | 0.0256 |
| BD vs D3 | 0.01430 | -0.1755 to 0.2041 | ns | >0.9999 |
| BD vs D4 | -0.1533 | -0.3431 to 0.03654 | ns | 0.1848 |
| BD vs D5 | 0.1419 | -0.04794 to 0.3317 | ns | 0.2589 |
| Amplitude SD/D | | | | |
| BD vs D1 | 0.09790 | -0.1429 to 0.3387 | ns | 0.8343 |
| BD vs D2 | 0.3774 | 0.1366 to 0.6182 | *** | 0.0003 |
| BD vs D3 | -0.1851 | -0.4259 to 0.05569 | ns | 0.2237 |
| BD vs D4 | -0.07110 | -0.3119 to 0.1697 | ns | 0.9515 |
| BD vs D5 | -0.2091 | -0.4499 to 0.03169 | ns | 0.1237 |
| Acrophase SD/L | | | | |
| BD vs D1 | 0.9000 | 0.04727 to 1.753 | * | 0.0324 |
| BD vs D2 | -0.4300 | -1.283 to 0.4227 | ns | 0.6850 |
| BD vs D3 | 0.08000 | -0.7727 to 0.9327 | ns | 0.9998 |
| BD vs D4 | 0.03000 | -0.8227 to 0.8827 | ns | >0.9999 |
| BD vs D5 | 0.4300 | -0.4227 to 1.283 | ns | 0.6850 |
| Acrophase SD/D | | | | |
| BD vs D1 | 0.9900 | -0.1217 to 2.102 | ns | 0.1072 |
| BD vs D2 | -1.050 | -2.162 to 0.06167 | ns | 0.0744 |
| BD vs D3 | 0.4800 | -0.6317 to 1.592 | ns | 0.7968 |
| BD vs D4 | 0.5200 | -0.5917 to 1.632 | ns | 0.7374 |
| BD vs D5 | -0.8300 | -1.942 to 0.2817 | ns | 0.2521 |

,

**Table 5: Statistics for the Non-Parametric Circadian Rhythm Analysis**

One-way RM ANOVA were performed to compare groups within the SD/L and SD/D conditions, as well as between equal subgroups of 12 subjects. All ANOVA tests were followed by Benjamini–Hochberg FDR correction for multiple comparisons and uncorrected Fisher’s LSD applied where appropriate.

| **L5** |  |  |  | |  |  | |  |  |  |
| --- | --- | --- | --- | --- | --- | --- | --- | --- | --- | --- |
| Number of families | 1 |  |  | |  |  | |  |  |  |
| Number of comparisons per family | 4 |  |  | |  |  | |  |  |  |
| Q | 0.1 |  |  | |  |  | |  |  |  |
| **Original FDR method of**  **Benjamini and Hochberg** | Mean Diff. | Discovery? | q value | Individual P Value | | |  |  |  |  |
| Mo/BD/L vs. Mo/BD/D | -140.4 | No | 0.8312 | | 0.2906 | A-D | |  |  |  |
| Mo/BD/L vs. Mo/AD/L | -28 | No | 0.8312 | | 0.8312 | A-B | |  |  |  |
| Mo/BD/D vs. Mo/AD/D | 54.11 | No | 0.8312 | | 0.6808 | D-E | |  |  |  |
| Mo/AD/L vs. Mo/AD/D | -58.33 | No | 0.8312 | | 0.6576 | B-E | |  |  |  |
| Test details | Mean 1 | Mean 2 | Mean Diff. | | SE of diff. | n1 | | n2 | t | DF |
| Mo/BD/L vs. Mo/BD/D | 812.8 | 953.2 | -140.4 | | 130 | 9 | | 9 | 1.081 | 24 |
| Mo/BD/L vs. Mo/AD/L | 812.8 | 840.8 | -28 | | 130 | 9 | | 9 | 0.2155 | 24 |
| Mo/BD/D vs. Mo/AD/D | 953.2 | 899.1 | 54.11 | | 130 | 9 | | 9 | 0.4164 | 24 |
| Mo/AD/L vs. Mo/AD/D | 840.8 | 899.1 | -58.33 | | 130 | 9 | | 9 | 0.4489 | 24 |
|  |  |  |  | |  |  | |  |  |  |
| **M10** |  |  |  | |  |  | |  |  |  |
| Mo/BD/L vs. Mo/BD/D | 4413 | No | 0.6642 | | 0.4982 | A-D | |  |  |  |
| Mo/BD/L vs. Mo/AD/L | 12586 | No | 0.2856 | | 0.0714 | A-B | |  |  |  |
| Mo/BD/D vs. Mo/AD/D | 1700 | No | 0.7862 | | 0.7862 | D-E | |  |  |  |
| Mo/AD/L vs. Mo/AD/D | -6473 | No | 0.4924 | | 0.2462 | B-E | |  |  |  |
| Test details | Mean 1 | Mean 2 | Mean Diff. | | SE of diff. | n1 | | n2 | t | DF |
| Mo/BD/L vs. Mo/BD/D | 41821 | 37409 | 4413 | | 6252 | 10 | | 10 | 0.7058 | 9 |
| Mo/BD/L vs. Mo/AD/L | 41821 | 29236 | 12586 | | 6160 | 10 | | 10 | 2.043 | 9 |
| Mo/BD/D vs. Mo/AD/D | 37409 | 35709 | 1700 | | 6082 | 10 | | 10 | 0.2795 | 9 |
| Mo/AD/L vs. Mo/AD/D | 29236 | 35709 | -6473 | | 5219 | 10 | | 10 | 1.24 | 9 |
|  |  |  |  | |  |  | |  |  |  |
| **RA** |  |  |  | |  |  | |  |  |  |
| Mo/BD/L vs. Mo/BD/D | 0.0011 | No | 0.8642 | | 0.8642 | A-D | |  |  |  |
| Mo/BD/L vs. Mo/AD/L | 0.0125 | No | 0.7869 | | 0.1967 | A-B | |  |  |  |
| Mo/BD/D vs. Mo/AD/D | 0.0029 | No | 0.8642 | | 0.7788 | D-E | |  |  |  |
| Mo/AD/L vs. Mo/AD/D | -0.0085 | No | 0.8642 | | 0.4551 | B-E | |  |  |  |
| Test details | Mean 1 | Mean 2 | Mean Diff. | | SE of diff. | n1 | | n2 | t | DF |
| Mo/BD/L vs. Mo/BD/D | 0.9551 | 0.954 | 0.0011 | | 0.006249 | 10 | | 10 | 0.176 | 9 |
| Mo/BD/L vs. Mo/AD/L | 0.9551 | 0.9426 | 0.0125 | | 0.008966 | 10 | | 10 | 1.394 | 9 |
| Mo/BD/D vs. Mo/AD/D | 0.954 | 0.9511 | 0.0029 | | 0.01002 | 10 | | 10 | 0.2894 | 9 |
| Mo/AD/L vs. Mo/AD/D | 0.9426 | 0.9511 | -0.0085 | | 0.01089 | 10 | | 10 | 0.7806 | 9 |
| **IV** |  |  |  | |  |  | |  |  |  |
| Mo/BD/L vs. Mo/AD/L | 0.4554 | **Yes** | **0.0651** | | **0.0324** | A-B | |  |  |  |
| Mo/BD/D vs. Mo/AD/D | -0.2478 | No | 0.144 | | 0.144 | D-E | |  |  |  |
| Mo/BD/L vs. Mo/BD/D | 0.4692 | **Yes** | **0.0651** | | **0.0325** | A-D | |  |  |  |
| Mo/AD/L vs. Mo/AD/D | -0.234 | **Yes** | **0.0925** | | **0.0694** | B-E | |  |  |  |
| Test details | Mean 1 | Mean 2 | Mean Diff. | | SE of diff. | n1 | | n2 | t | DF |
| Mo/BD/L vs. Mo/AD/L | 1.294 | 0.839 | 0.4554 | | 0.1802 | 10 | | 10 | 2.527 | 9 |
| Mo/BD/D vs. Mo/AD/D | 0.8252 | 1.073 | -0.2478 | | 0.1549 | 10 | | 10 | 1.6 | 9 |
| Mo/BD/L vs. Mo/BD/D | 1.294 | 0.8252 | 0.4692 | | 0.1859 | 10 | | 10 | 2.524 | 9 |
| Mo/AD/L vs. Mo/AD/D | 0.839 | 1.073 | -0.234 | | 0.1135 | 10 | | 10 | 2.061 | 9 |

| **Uncorrected Fisher's LSD** | Mean diff, | 95,00% CI of diff, | Below threshold? | Summary | Individual P Value |
| --- | --- | --- | --- | --- | --- |
| Mo/BD/L vs. Mo/AD/L | 0.4654 | 0.06438 to 0.8664 | Yes | * | 0.0276 |
| Mo/BD/D vs. Mo/AD/D | -0.2478 | -0.5981 to 0.1025 | No | ns | 0.1440 |
| Mo/BD/L vs. Mo/BD/D | 0.4692 | 0.04873 to 0.8897 | Yes | * | 0.0325 |
| Mo/AD/L vs. Mo/AD/D | -0.2440 | -0.4934 to 0.005450 | No | ns | 0.0542 |

**Table 6: Statistics for the Stanford Sleepiness Scale assessment**

A) RM ANOVA comparing groups within the SD/L or SD/D conditions.

B) RM ANOVA comparing the equal SD/L and SD/D groups of 12 subjects.

All ANOVA tests were followed by FDR correction for multiple comparisons using the Benjamini-Hochberg method and uncorrected Fisher’s LSD applied where appropriate.

**(A)**

| Number of families | 1 |  |  |  |  |  |  |  |
| --- | --- | --- | --- | --- | --- | --- | --- | --- |
| Number of comparisons per family | 8 |  |  |  |  |  |  |  |
| Q | 0.1 |  |  |  |  |  |  |  |
| **Original FDR method  of Benjamini and Hochberg** |  |  |  |  |  |  |  |  |
|  | Mean Diff. | Discovery? | q value | Individual P Value |  |  |  |  |
| BM/L vs. D1M/L | -2.028 | **Yes** | **<0.0001** | **<0.0001** | A-C |  |  |  |
| BM/L vs. RM/L | 0.1111 | No | 0.6254 | 0.6254 | A-E |  |  |  |
| D1M/L vs. RM/L | 2.139 | **Yes** | **<0.0001** | **<0.0001** | C-E |  |  |  |
| BE/L vs. D1E/L | -1.639 | **Yes** | **0.0004** | **0.0003** | B-D |  |  |  |
| BM/D vs. D1M/D | -2.208 | **Yes** | **0.0001** | **<0.0001** | G-I |  |  |  |
| BM/D vs. RM/D | 0.2500 | No | 0.2562 | 0.2562 | G-K |  |  |  |
| D1M/D vs. RM/D | 2.458 | **Yes** | **<0.0001** | **<0.0001** | I-K |  |  |  |
| BE/D vs. D1E/D | -2.083 | **Yes** | **<0.0001** | **<0.0001** | H-J |  |  |  |
| Test details | Mean 1 | Mean 2 | Mean Diff. | SE of diff. | n1 | n2 | t | DF |
| BM/L vs. D1M/L | 2.444 | 4.472 | -2.028 | 0.2040 | 18 | 18 | 9.939 | 17 |
| BM/L vs. RM/L | 2.444 | 2.333 | 0.1111 | 0.2234 | 18 | 18 | 0.4973 | 17 |
| D1M/L vs. RM/L | 4.472 | 2.333 | 2.139 | 0.2093 | 18 | 18 | 10.22 | 17 |
| BE/L vs. D1E/L | 2.722 | 4.361 | -1.639 | 0.3656 | 18 | 18 | 4.483 | 17 |
| BM/D vs. D1M/D | 2.875 | 5.083 | -2.208 | 0.3667 | 12 | 12 | 6.136 | 11 |
| BM/D vs. RM/D | 2.875 | 2.625 | 0.2500 | 0.2087 | 12 | 12 | 1.198 | 11 |
| D1M/D vs. RM/D | 5.125 | 2.625 | 2.458 | 0.2538 | 12 | 12 | 9.560 | 11 |
| BE/D vs. D1E/D | 2.583 | 4.667 | -2.083 | 0.3067 | 12 | 12 | 6.793 | 11 |

| Number of families | 1 |  |  | | |  |  |  |  |  |
| --- | --- | --- | --- | --- | --- | --- | --- | --- | --- | --- |
| Number of comparisons per family | 4 |  |  | | |  |  |  |  |  |
| Alpha | 0.05 |  |  | | |  |  |  |  |  |
|  |  |  |  | | |  |  |  |  |  |
| **Uncorrected Fisher's LSD** | Mean diff, | 95.00% CI of diff, | Below threshold? | | | Summary | Individual P Value |  |  |  |
| BM/L vs. D1M/L | -2.028 | -2.458 to -1.597 | Yes | | | **** | <0.0001 |  |  |  |
| BM/L vs. RM/L | 0.1111 | -0.3603 to 0.5825 | No | | | ns | 0.6254 |  |  |  |
| D1M/L vs. RM/L | 2.139 | 1.697 to 2.580 | Yes | | | **** | <0.0001 |  |  |  |
| BE/L vs. D1E/L | -1.639 | -2.410 to -0.8675 | Yes | | | *** | 0.0003 |  |  |  |
| BM/D vs. D1M/D | -2.208 | -3.015 to -1.402 | | Yes |  | **** | <0.0001 |  |  |  |
| BM/D vs. RM/D | 0.2500 | -0.2094 to 0.7094 | | No |  | ns | 0.2562 |  |  |  |
| D1M/D vs. RM/D | 2.458 | 1.892 to 3.024 | | Yes |  | **** | <0.0001 |  |  |  |
| BE/D vs. D1E/D | -2.083 | -2.758 to -1.408 | | Yes |  | **** | <0.0001 |  |  |  |

**(B)**

| Number of families | 1 |  |  |  |  |  |  |  |
| --- | --- | --- | --- | --- | --- | --- | --- | --- |
| Number of comparisons per family | 5 |  |  |  |  |  |  |  |
| Q | 0.1 |  |  |  |  |  |  |  |
| **Original FDR method  of Benjamini and Hochberg** | Mean Diff. | Discovery? | q value | Individual P Value |  |  |  |  |
| BM/L vs. BM/D | -0.25 | No | 0.8724 | 0.5459 | A-G |  |  |  |
| BE/L vs. BE/D | -0.08333 | No | 0.8724 | 0.7401 | B-H |  |  |  |
| D1M/L vs. D1M/D | -0.7083 | **Yes** | **0.0688** | **0.0138** | C-I |  |  |  |
| D1E/L vs. D1E/D | -0.08333 | No | 0.8724 | 0.8724 | D-J |  |  |  |
| RM/L vs. RM/D | -0.3333 | No | 0.4152 | 0.1661 | E-K |  |  |  |
| Test details | Mean 1 | Mean 2 | Mean Diff. | SE of diff. | n1 | n2 | t | DF |
| BM/L vs. BM/D | 2.625 | 2.875 | -0.25 | 0.4012 | 12 | 12 | 0.6231 | 11 |
| BE/L vs. BE/D | 2.5 | 2.583 | -0.08333 | 0.2449 | 12 | 12 | 0.3403 | 11 |
| D1M/L vs. D1M/D | 4.417 | 5.125 | -0.7083 | 0.242 | 12 | 12 | 2.927 | 11 |
| D1E/L vs. D1E/D | 4.583 | 4.667 | -0.08333 | 0.5069 | 12 | 12 | 0.1644 | 11 |
| RM/L vs. RM/D | 2.292 | 2.625 | -0.3333 | 0.2247 | 12 | 12 | 1.483 | 11 |

| Number of families | 1 |  |  |  |  |  |
| --- | --- | --- | --- | --- | --- | --- |
| Number of comparisons per family | 5 |  |  |  |  |  |
| Alpha | 0.05 |  |  |  |  |  |
|  |  |  |  |  |  |  |
| **Uncorrected Fisher's LSD** | Mean diff, | 95.00% CI of diff, | Below threshold? | Summary | Individual P Value |  |
| BM/L vs. BM/D | -0.2500 | -1.133 to 0.6331 | No | ns | 0.5459 |  |
| BE/L vs. BE/D | -0.08333 | -0.6223 to 0.4557 | No | ns | 0.7401 |  |
| D1M/L vs. D1M/D | -0.7083 | -1.241 to -0.1757 | Yes | * | 0.0138 |  |
| D1E/L vs. D1E/D | -0.08333 | -1.199 to 1.032 | No | ns | 0.8724 |  |
| RM/L vs. RM/D | -0.3333 | -0.8280 to 0.1613 | No | ns | 0.1661 |  |

**Table 7: Statistics for the positive PANAS assessment**

A) RM ANOVA comparing groups within the SD/L or SD/D conditions.

B) RM ANOVA comparing the equal SD/L and SD/D groups of 12 subjects.

All ANOVA tests were followed by FDR correction for multiple comparisons using the Benjamini-Hochberg method and uncorrected Fisher’s LSD applied where appropriate.

**(A)**

| Number of families | 1 | |  | |  |  |  |  |  |  |
| --- | --- | --- | --- | --- | --- | --- | --- | --- | --- | --- |
| Number of comparisons per family | 8 | |  | |  |  |  |  |  |  |
| Q | 0.1 | |  | |  |  |  |  |  |  |
| **Original FDR method of Benjamini and Hochberg** | Mean Diff. | Discovery? | | | q value | Individual P Value |  |  |  |  |
| BM/L vs. D1M/L | 8.333 | | **Yes** | **<0.0001** | | **<0.0001** | A-B |  |  |  |
| BM/L vs. RM/L | 2.278 | | **Yes** | **0.0327** | | **0.0327** | A-C |  |  |  |
| D1M/L vs. RM/L | -6.056 | | **Yes** | **0.0001** | | **<0.0001** | B-C |  |  |  |
| BE/L vs. D1E/L | 7.444 | | **Yes** | **0.0002** | | **0.0001** | I-J |  |  |  |
| BM/D vs. D1M/D | 10.67 | | **Yes** | **0.0001** | | **<0.0001** | E-F |  |  |  |
| BM/D vs. RM/D | 3.333 | | No | 0.232 | | 0.232 | E-G |  |  |  |
| D1M/D vs. RM/D | -7.333 | | **Yes** | **0.0073** | | **0.0046** | F-G |  |  |  |
| BE/D vs. D1E/D | 5.5 | | **Yes** | **0.009** | | **0.0068** | L-M |  |  |  |
| Test details | Mean 1 | | Mean 2 | Mean Diff. | | SE of diff. | n1 | n2 | t | DF |
| BM/L vs. D1M/L | 26.94 | | 18.61 | | 8.333 | 1.042 | 18 | 18 | 8.000 | 17 |
| BM/L vs. RM/L | 26.94 | | 24.67 | | 2.278 | 0.9795 | 18 | 18 | 2.325 | 17 |
| D1M/L vs. RM/L | 18.61 | | 24.67 | | -6.056 | 1.150 | 18 | 18 | 5.264 | 17 |
| BE/L vs. D1E/L | 24.50 | | 17.06 | | 7.444 | 1.509 | 18 | 18 | 4.935 | 17 |
| BM/D vs. D1M/D | 26 | | 15.33 | | 10.67 | 1.479 | 12 | 12 | 7.213 | 11 |
| BM/D vs. RM/D | 26 | | 22.67 | | 3.333 | 2.635 | 12 | 12 | 1.265 | 11 |
| D1M/D vs. RM/D | 15.33 | | 22.67 | | -7.333 | 2.068 | 12 | 12 | 3.546 | 11 |
| BE/D vs. D1E/D | 20 | | 14.5 | | 5.5 | 1.654 | 12 | 12 | 3.326 | 11 |

| Number of families | 1 |  |  |  |  |  |
| --- | --- | --- | --- | --- | --- | --- |
| Number of comparisons per family | 8 |  |  |  |  |  |
| Alpha | 0.05 |  |  |  |  |  |
|  |  |  |  |  |  |  |
| **Uncorrected Fisher's LSD** | Mean diff, | 95.00% CI of diff, | Below threshold? | Summary | Individual P Value |  |
| BM/L vs. D1M/L | 8.333 | 6.136 to 10.53 | Yes | **** | <0.0001 |  |
| BM/L vs. RM/L | 2.278 | 0.2111 to 4.344 | Yes | * | 0.0327 |  |
| D1M/L vs. RM/L | -6.056 | -8.483 to -3.628 | Yes | **** | <0.0001 |  |
| BE/L vs. D1E/L | 7.444 | 4.262 to 10.63 | Yes | *** | 0.0001 |  |
| BM/D vs. D1M/D | 10.67 | 7.412 to 13.92 | Yes | **** | <0.0001 |  |
| BM/D vs. RM/D | 3.333 | -2.467 to 9.133 | No | ns | 0.2320 |  |
| D1M/D vs. RM/D | -7.333 | -11.89 to -2.781 | Yes | ** | 0.0046 |  |
| BE/D vs. D1E/D | 5.500 | 1.860 to 9.140 | Yes | ** | 0.0068 |  |

**(B)**

| Number of families | 1 |  |  | |  | |  | |  | |  | |  | |
| --- | --- | --- | --- | --- | --- | --- | --- | --- | --- | --- | --- | --- | --- | --- |
| Number of comparisons per family | 5 |  |  | |  | |  | |  | |  | |  | |
| Q | 0.1 |  |  | |  | |  | |  | |  | |  | |
| **Original FDR method of Benjamini and Hochberg** | Mean Diff. | Discovery? | | q value | | Individual P Value | |  | |  | |  | |  |
| BM/L vs. BM/D | -1.167 | No | 0.908 | | 0.6354 | | A-E | |  | |  | |  | |
| D1M/L vs. D1M/D | 2.083 | No | 0.908 | | 0.4053 | | B-F | |  | |  | |  | |
| D1E/L vs. D1E/D | 0.5 | No | 0.908 | | 0.8559 | | J-M | |  | |  | |  | |
| RM/L vs. RM/D | 0.4167 | No | 0.908 | | 0.908 | | C-G | |  | |  | |  | |
| BE/L vs. BE/D | 1.25 | No | 0.908 | | 0.5403 | | I-L | |  | |  | |  | |
| Test details | Mean 1 | Mean 2 | Mean Dif | | SE of diff. | | n1 | | n2 | | t | | DF | |
| BM/L vs. BM/D | 24.83 | 26 | -1.167 | | 2.393 | | 12 | | 12 | | 0.4876 | | 11 | |
| D1M/L vs. D1M/D | 17.42 | 15.33 | 2.083 | | 2.407 | | 12 | | 12 | | 0.8654 | | 11 | |
| D1E/L vs. D1E/D | 15 | 14.5 | 0.5 | | 2.69 | | 12 | | 12 | | 0.1859 | | 11 | |
| RM/L vs. RM/D | 23.08 | 22.67 | 0.4167 | | 3.524 | | 12 | | 12 | | 0.1182 | | 11 | |
| BE/L vs. BE/D | 21.25 | 20 | 1.25 | | 1.978 | | 12 | | 12 | | 0.6321 | | 11 | |

**Table 8: Statistics for the negative PANAS assessment**

A) RM ANOVA comparing groups within the SD/L or SD/D conditions.

B) RM ANOVA comparing the equal SD/L and SD/D groups of 12 subjects.

All ANOVA tests were followed by FDR correction for multiple comparisons using the Benjamini-Hochberg method and uncorrected Fisher’s LSD applied where appropriate.

**(A)**

| Number of families | 1 |  |  |  |  |  |  |  |
| --- | --- | --- | --- | --- | --- | --- | --- | --- |
| Number of comparisons per family | 8 |  |  |  |  |  |  |  |
| Q | 0.1 |  |  |  |  |  |  |  |
|  |  |  |  |  |  |  |  |  |
| **Original FDR method of Benjamini and Hochberg** | Mean Diff. | Discovery? | q value | Individual P Value |  |  |  |  |
| BM/L vs. D1M/L | -0.7778 | No | 0.3508 | 0.3508 | A-B |  |  |  |
| BM/L vs. RM/L | 1.056 | No | 0.2870 | 0.1435 | A-C |  |  |  |
| D1M/L vs. RM/L | 1.833 | No | 0.2133 | 0.0533 | B-C |  |  |  |
| BE/L vs. D1E/L | 0.6667 | No | 0.3400 | 0.2550 | I-J |  |  |  |
| BM/D vs. D1M/D | -1.25 | No | 0.2657 | 0.2061 | E-F |  |  |  |
| BM/D vs. RM/D | 2.667 | **Yes** | **0.0352** | **0.0176** | E-G |  |  |  |
| D1M/D vs. RM/D | 3.917 | **Yes** | **0.0205** | **0.0051** | F-G |  |  |  |
| BE/D vs. D1E/D | 1 | No | 0.2657 | 0.2657 | L-M |  |  |  |
| Test details | Mean 1 | Mean 2 | Mean Diff. | SE of diff. | n1 | n2 | t | DF |
| BM/L vs. D1M/L | 11.33 | 12.08 | -0.75 | 0.4787 | 12 | 12 | 1.567 | 11 |
| BM/L vs. RM/L | 11.33 | 10.58 | 0.75 | 0.4943 | 12 | 12 | 1.517 | 11 |
| D1M/L vs. RM/L | 12.08 | 10.58 | 1.5 | 0.7638 | 12 | 12 | 1.964 | 11 |
| BE/L vs. D1E/L | 11.25 | 11.25 | 0 | 0.5222 | 12 | 12 | 0 | 11 |
| BM/D vs. D1M/D | 13.83 | 15.08 | -1.25 | 0.9303 | 12 | 12 | 1.344 | 11 |
| BM/D vs. RM/D | 13.83 | 11.17 | 2.667 | 0.9561 | 12 | 12 | 2.789 | 11 |
| D1M/D vs. RM/D | 15.08 | 11.17 | 3.917 | 1.125 | 12 | 12 | 3.483 | 11 |
| BE/D vs. D1E/D | 14.5 | 13.5 | 1 | 0.8528 | 12 | 12 | 1.173 | 11 |

| Number of families | 1 |  |  |  |  |  |
| --- | --- | --- | --- | --- | --- | --- |
| Number of comparisons per family | 4 |  |  |  |  |  |
| Alpha | 0.05 |  |  |  |  |  |
|  |  |  |  |  |  |  |
| **Uncorrected Fisher's LSD** | Mean diff, | 95.00% CI of diff, | Below threshold? | Summary | Individual P Value |  |
| BM/D vs. D1M/D | -1.250 | -3.298 to 0.7977 | No | ns | 0.2061 | E-F |
| BM/D vs. RM/D | 2.667 | 0.5623 to 4.771 | Yes | * | 0.0176 | E-G |
| D1M/D vs. RM/D | 3.917 | 1.442 to 6.392 | Yes | ** | 0.0051 | F-G |
| BE/D vs. D1E/D | 1.000 | -0.8770 to 2.877 | No | ns | 0.2657 | L-M |

**(B)**

| Number of families | 1 |  |  |  |  |  |  |  |
| --- | --- | --- | --- | --- | --- | --- | --- | --- |
| Number of comparisons per family | 5 |  |  |  |  |  |  |  |
| Q | 0.1 |  |  |  |  |  |  |  |
|  |  |  |  |  |  |  |  |  |
| **Original FDR method of Benjamini and Hochberg** | Mean Diff. | Discovery? | q value | Individual P Value |  |  |  |  |
| BM/L vs. BM/D | -2.5 | No | 0.1589 | 0.1272 | A-E |  |  |  |
| D1M/L vs. D1M/D | -3 | No | 0.1589 | 0.0892 | B-F |  |  |  |
| D1E/L vs. D1E/D | -2.25 | No | 0.1589 | 0.1151 | J-M |  |  |  |
| RM/L vs. RM/D | -0.5833 | No | 0.2534 | 0.2534 | C-G |  |  |  |
| BE/L vs. BE/D | -3.25 | No | 0.1235 | 0.0247 | I-L |  |  |  |
| Test details | Mean 1 | Mean 2 | Mean Diff. | SE of diff. | n1 | n2 | t | DF |
| BM/L vs. BM/D | 11.33 | 13.83 | -2.5 | 1.515 | 12 | 12 | 1.65 | 11 |
| D1M/L vs. D1M/D | 12.08 | 15.08 | -3 | 1.61 | 12 | 12 | 1.864 | 11 |
| D1E/L vs. D1E/D | 11.25 | 13.5 | -2.25 | 1.315 | 12 | 12 | 1.711 | 11 |
| RM/L vs. RM/D | 10.58 | 11.17 | -0.5833 | 0.484 | 12 | 12 | 1.205 | 11 |
| BE/L vs. BE/D | 11.25 | 14.5 | -3.25 | 1.25 | 12 | 12 | 2.6 | 11 |

|  |
| --- |

**Table 9: Statistics for Simple Response Time Task score assessment**

A) RM ANOVA comparing groups within the SD/L or SD/D conditions.

B) RM ANOVA comparing the equal SD/L and SD/D groups of 12 subjects.

All ANOVA tests were followed by FDR correction for multiple comparisons using the Benjamini-Hochberg method and uncorrected Fisher’s LSD applied where appropriate.

**(A)**

| Number of families | 1 |  | |  |  |  |  |  |  |
| --- | --- | --- | --- | --- | --- | --- | --- | --- | --- |
| Number of comparisons per family | 8 |  | |  |  |  |  |  |  |
| Q | 0.1 |  | |  |  |  |  |  |  |
|  |  |  | |  |  |  |  |  |  |
| **Original FDR method of Benjamini and Hochberg** | Mean Diff. | Discovery? | q value | | Individual P Value |  |  |  |  |
| BM/L vs. D1M/L | -17.08 | **Yes** | **0.0937** | | **0.0234** | A-C |  |  |  |
| BM/L vs. RM/L | -7.889 | No | 0.3339 | | 0.3339 | A-E |  |  |  |
| D1M/L vs. RM/L | 9.194 | No | 0.1834 | | 0.1375 | C-E |  |  |  |
| BE/L vs. D1E/L | -11.67 | No | 0.1552 | | 0.0776 | B-D |  |  |  |
| BM/D vs. D1M/D | -32.63 | **Yes** | **0.0019** | | **0.0014** | F-H |  |  |  |
| BM/D vs. RM/D | -5.292 | No | 0.3185 | | 0.3185 | F-J |  |  |  |
| D1M/D vs. RM/D | 27.33 | **Yes** | **0.0006** | | **0.0003** | H-J |  |  |  |
| BE/D vs. D1E/D | -26.83 | **Yes** | **0.0001** | | **<0.0001** | G-I |  |  |  |
| Test details | Mean 1 | Mean 2 | Mean Diff. | | SE of diff. | n1 | n2 | t | DF |
| BM/L vs. D1M/L | 284.9 | 301.9 | -17.08 | | 6.861 | 18 | 18 | 2.490 | 17 |
| BM/L vs. RM/L | 284.9 | 292.8 | -7.889 | | 7.932 | 18 | 18 | 0.9946 | 17 |
| D1M/L vs. RM/L | 301.9 | 292.8 | 9.194 | | 5.900 | 18 | 18 | 1.559 | 17 |
| BE/L vs. D1E/L | 277.5 | 289.2 | -11.67 | | 6.212 | 18 | 18 | 1.878 | 17 |
| BM/D vs. D1M/D | 278.5 | 311.1 | -32.63 | | 7.734 | 12 | 12 | 4.219 | 11 |
| BM/D vs. RM/D | 278.5 | 283.8 | -5.292 | | 5.065 | 12 | 12 | 1.045 | 11 |
| D1M/D vs. RM/D | 311.1 | 283.8 | 27.33 | | 5.308 | 12 | 12 | 5.149 | 11 |
| BE/D vs. D1E/D | 277.9 | 304.8 | -26.83 | | 3.998 | 12 | 12 | 6.711 | 11 |

| Number of families | 1 |  | |  | |  |  |  |
| --- | --- | --- | --- | --- | --- | --- | --- | --- |
| Number of comparisons per family | 4 |  | |  | |  |  |  |
| Alpha | 0.05 |  | |  | |  |  |  |
|  |  |  | |  | |  |  |  |
| **Uncorrected Fisher's LSD** | Mean diff, | 95.00% CI of diff, | | Below threshold? | | Summary | Individual P Value |  |
| BM/L vs. D1M/L | -17.08 | -31.56 to -2.609 | | Yes | | * | 0.0234 |  |
| BM/L vs. RM/L | -7.889 | -24.62 to 8.846 | | No | | ns | 0.3339 |  |
| D1M/L vs. RM/L | 9.194 | -3.252 to 21.64 | | No | | ns | 0.1375 |  |
| BE/L vs. D1E/L | -11.67 | -24.77 to 1.439 | | No | | ns | 0.0776 |  |
| BM/D vs. D1M/D | -32.63 | -49.65 to -15.60 |  | | Yes | ** | 0.0014 |  |
| BM/D vs. RM/D | -5.292 | -16.44 to 5.855 |  | | No | ns | 0.3185 |  |
| D1M/D vs. RM/D | 27.33 | 15.65 to 39.02 |  | | Yes | *** | 0.0003 |  |
| BE/D vs. D1E/D | -26.83 | -35.63 to -18.03 |  | | Yes | **** | <0.0001 |  |

**(B)**

| Number of families | 1 | |  |  |  |  |  |  |  |
| --- | --- | --- | --- | --- | --- | --- | --- | --- | --- |
| Number of comparisons per family | 5 | |  |  |  |  |  |  |  |
| Q | 0.1 | |  |  |  |  |  |  |  |
|  |  | |  |  |  |  |  |  |  |
| **Original FDR method of Benjamini and Hochberg** | | Mean Diff. | Discovery? | q value | Individual P Value |  |  |  |  |
| BM/L vs. BM/D | 7.625 | | No | 0.4413 | 0.2648 | A-F |  |  |  |
| BE/L vs. BE/D | -0.5000 | | No | 0.9270 | 0.9270 | B-G |  |  |  |
| D1M/L vs. D1M/D | -20.33 | | **Yes** | **0.0206** | **0.0041** | C-H |  |  |  |
| D1E/L vs. D1E/D | -12.33 | | No | 0.4132 | 0.1653 | D-I |  |  |  |
| RM/L vs. RM/D | 5.875 | | No | 0.5681 | 0.4545 | E-J |  |  |  |
| Test details | Mean 1 | | Mean 2 | Mean Diff. | SE of diff. | n1 | n2 | t | DF |
| BM/L vs. BM/D | 286.1 | | 278.5 | 7.625 | 6.489 | 12 | 12 | 1.175 | 11 |
| BE/L vs. BE/D | 277.4 | | 277.9 | -0.5000 | 5.334 | 12 | 12 | 0.0937 | 11 |
| D1M/L vs. D1M/D | 290.8 | | 311.1 | -20.33 | 5.636 | 12 | 12 | 3.608 | 11 |
| D1E/L vs. D1E/D | 292.4 | | 304.8 | -12.33 | 8.298 | 12 | 12 | 1.486 | 11 |
| RM/L vs. RM/D | 289.7 | | 283.8 | 5.875 | 7.578 | 12 | 12 | 0.7753 | 11 |

| Number of families | 1 |  |  |  |  |  |
| --- | --- | --- | --- | --- | --- | --- |
| Number of comparisons per family | 5 |  |  |  |  |  |
| Alpha | 0.05 |  |  |  |  |  |
|  |  |  |  |  |  |  |
| **Uncorrected Fisher's LSD** | Mean diff, | 95.00% CI of diff, | Below threshold? | Summary | Individual P Value |  |
| BM/L vs. BM/D | 7.625 | -6.657 to 21.91 | No | ns | 0.2648 |  |
| BE/L vs. BE/D | -0.5000 | -12.24 to 11.24 | No | ns | 0.9270 |  |
| D1M/L vs. D1M/D | -20.33 | -32.74 to -7.928 | Yes | ** | 0.0041 |  |
| D1E/L vs. D1E/D | -12.33 | -30.60 to 5.930 | No | ns | 0.1653 |  |
| RM/L vs. RM/D | 5.875 | -10.80 to 22.55 | No | ns | 0.4545 |  |

**Table 10: Statistics for Glycaemia Assessment**

A) RM ANOVA comparing groups within the SD/L or SD/D conditions before and after a meal.

B) RM ANOVA comparing the equal SD/L and SD/D groups of 12 subjects before and after a meal.

All ANOVA tests were followed by FDR correction for multiple comparisons using the Benjamini-Hochberg method and uncorrected Fisher’s LSD applied where appropriate.

**(A)**

**BEFORE meal**

| Number of families | 1 | |  |  |  |  |  |  |  |
| --- | --- | --- | --- | --- | --- | --- | --- | --- | --- |
| Number of comparisons per family | 6 | |  |  |  |  |  |  |  |
| Q | 0.1 | |  |  |  |  |  |  |  |
|  |  | |  |  |  |  |  |  |  |
| **Original FDR method of Benjamini and Hochberg** | | Mean Diff. | Discovery? | q value | Individual P Value |  |  |  |  |
| BM/L vs. D1M/L | | 0.01667 | No | >0.9999 | 0.9068 | A-B |  |  |  |
| BM/L vs. RM/L | | 0.25 | No | 0.1342 | 0.0224 | A-C |  |  |  |
| D1M/L vs. RM/L | | 0.2333 | No | 0.4731 | 0.1577 | B-C |  |  |  |
| BM/D vs. D1M/D | | -0.01667 | No | >0.9999 | 0.8837 | E-F |  |  |  |
| BM/D vs. RM/D | | 0 | No | >0.9999 | >0.9999 | E-G |  |  |  |
| D1M/D vs. RM/D | | 0.01667 | No | >0.9999 | 0.9194 | F-G |  |  |  |
| Test details | | Mean 1 | Mean 2 | Mean Diff. | SE of diff. | n1 | n2 | t | DF |
| BM/L vs. D1M/L | | 4.617 | 4.6 | 0.01667 | 0.1392 | 12 | 12 | 0.1198 | 11 |
| BM/L vs. RM/L | | 4.617 | 4.367 | 0.25 | 0.09415 | 12 | 12 | 2.655 | 11 |
| D1M/L vs. RM/L | | 4.6 | 4.367 | 0.2333 | 0.1539 | 12 | 12 | 1.516 | 11 |
| BM/D vs. D1M/D | | 4.742 | 4.758 | -0.01667 | 0.1114 | 12 | 12 | 0.1497 | 11 |
| BM/D vs. RM/D | | 4.742 | 4.742 | 0 | 0.1867 | 12 | 12 | 0 | 11 |
| D1M/D vs. RM/D | | 4.758 | 4.742 | 0.01667 | 0.1609 | 12 | 12 | 0.1036 | 11 |

**AFTER meal**

| Number of families | 1 |  |  |  |  |  |  |  |
| --- | --- | --- | --- | --- | --- | --- | --- | --- |
| Number of comparisons per family | 6 |  |  |  |  |  |  |  |
| Q | 0.1 |  |  |  |  |  |  |  |
| **Original FDR method of Benjamini and Hochberg** | Mean Diff. | Discovery? | q value | Individual P Value |  |  |  |  |
| BM/L vs. D1M/L | -0.675 | No | 0.2602 | 0.1221 | A-B |  |  |  |
| BM/L vs. RM/L | -0.01667 | No | 0.9622 | 0.9622 | A-C |  |  |  |
| D1M/L vs. RM/L | 0.6583 | No | 0.2938 | 0.1959 | B-C |  |  |  |
| BM/D vs. D1M/D | 0.15 | No | 0.8042 | 0.6701 | E-F |  |  |  |
| BM/D vs. RM/D | -0.725 | No | 0.1757 | 0.0293 | E-G |  |  |  |
| D1M/D vs. RM/D | -0.875 | No | 0.2602 | 0.1301 | F-G |  |  |  |
| Test details | Mean 1 | Mean 2 | Mean Diff. | SE of diff. | n1 | n2 | t | DF |
| BM/L vs. D1M/L | 5.633 | 6.308 | -0.675 | 0.403 | 12 | 12 | 1.675 | 11 |
| BM/L vs. RM/L | 5.633 | 5.65 | -0.01667 | 0.344 | 12 | 12 | 0.04846 | 11 |
| D1M/L vs. RM/L | 6.308 | 5.65 | 0.6583 | 0.4781 | 12 | 12 | 1.377 | 11 |
| BM/D vs. D1M/D | 5.908 | 5.758 | 0.15 | 0.3428 | 12 | 12 | 0.4376 | 11 |
| BM/D vs. RM/D | 5.908 | 6.633 | -0.725 | 0.2895 | 12 | 12 | 2.504 | 11 |
| D1M/D vs. RM/D | 5.758 | 6.633 | -0.875 | 0.5349 | 12 | 12 | 1.636 | 11 |

**(B)**

**BEFORE meal**

| Number of families | 1 |  |  |  |  |  |  |  |
| --- | --- | --- | --- | --- | --- | --- | --- | --- |
| Number of comparisons per family | 3 |  |  |  |  |  |  |  |
| Q | 0.1 |  |  |  |  |  |  |  |
| **Original FDR method of Benjamini and Hochberg** | Predicted (LS) mean diff. | Discovery? | q value | Individual P Value |  |  |  |  |
| BM/L vs. BM/D | -0.1636 | No | 0.3120 | 0.2080 | A-E |  |  |  |
| D1M/L vs. D1M/D | -0.1636 | No | 0.3409 | 0.3409 | B-F |  |  |  |
| RM/L vs. RM/D | -0.3545 | **Yes** | **0.0076** | 0.0025 | C-G |  |  |  |
| Test details | Mean 1 | Mean 2 | Mean diff, | SE of diff, | n1 | n2 | t | DF |
| BM/L vs. BM/D | 4.573 | 4.736 | -0.1636 | 0.1216 | 12 | 12 | 1.346 | 11 |
| D1M/L vs. D1M/D | 4.573 | 4.736 | -0.1636 | 0.1636 | 12 | 12 | 1.000 | 11 |
| RM/L vs. RM/D | 4.282 | 4.636 | -0.3545 | 0.08879 | 12 | 12 | 3.993 | 11 |

| **Uncorrected Fisher's LSD** | Mean diff, | 95.00% CI of diff, | Below threshold? | Summary | Individual P Value |  |
| --- | --- | --- | --- | --- | --- | --- |
| BM/L vs. BM/D | -0.1636 | -0.4345 to 0.1072 | No | ns | 0.2080 |  |
| D1M/L vs. D1M/D | -0.1636 | -0.5282 to 0.2010 | No | ns | 0.3409 |  |
| RM/L vs. RM/D | -0.3545 | -0.5524 to -0.1567 | Yes | ** | 0.0025 |  |

**AFTER meal**

| Number of families | 1 |  |  |  |  |  |  |  |
| --- | --- | --- | --- | --- | --- | --- | --- | --- |
| Number of comparisons per family | 3 |  |  |  |  |  |  |  |
| Q | 0.1 |  |  |  |  |  |  |  |
| **Original FDR method of Benjamini and Hochberg** | Mean Diff. | Discovery? | q value | Individual P Value |  |  |  |  |
| BM/L vs. BM/D | -0.275 | No | 0.4814 | 0.4814 | A-E |  |  |  |
| D1M/L vs. D1M/D | 0.55 | No | 0.3270 | 0.2180 | B-F |  |  |  |
| RM/L vs. RM/D | -0.9833 | No | 0.2477 | 0.0826 | C-G |  |  |  |
| Test details | Mean 1 | Mean 2 | Mean Diff. | SE of diff. | n1 | n2 | t | DF |
| BM/L vs. BM/D | 5.633 | 5.908 | -0.275 | 0.4204 | 12 | 12 | 0.6541 | 55 |
| D1M/L vs. D1M/D | 6.308 | 5.758 | 0.55 | 0.4204 | 12 | 12 | 1.308 | 55 |
| RM/L vs. RM/D | 5.65 | 6.633 | -0.9833 | 0.4204 | 12 | 12 | 2.339 | 55 |

**Table 11: Statistics for Visual Analogue Scale Assessment of Satiety and Sweet, Salty, Piquant, and Greasy Taste Preferences** Using RM ANOVA within SD/L and SD/D conditions, with post-hoc tests (Benjamini–Hochberg FDR and Uncorrected Fisher’s LSD when applicable).

**SATIETY**

Satiety differed significantly across time points in both sessions:

SD/L: (F(5.643, 95.92) = 37.14, p < 0.0001, R² = 0.6860)

SD/D (F(5.206, 57.26) = 32.48, p < 0.0001, R² = 0.7470)

| Number of families | 1 |  |  |  |  |  | |  | |  |
| --- | --- | --- | --- | --- | --- | --- | --- | --- | --- | --- |
| Number of comparisons per family | 22 |  |  |  |  |  | |  | |  |
| Q | 0.1 |  |  |  |  |  | |  | |  |
| **Original FDR method of  Benjamini and Hochberg** | Mean Diff. | Discovery? | q value | Individual  P Value |  |  |  | |  |  |
| BBB/L vs. BBL/L | -1.139 | No | 0.1003 | 0.0729 | A-C |  |  |  |  |  |
| BBB/L vs. BBD/L | -2.033 | **Yes** | **0.0032** | **0.0005** | A-E |  |  |  |  |  |
| BBL/L vs. BBD/L | -0.8944 | **Yes** | **0.0300** | **0.0164** | C-E |  |  |  |  |  |
| BBB/L vs. D1BB/L | -2.400 | **Yes** | **0.0032** | **0.0006** | A-M |  |  |  |  |  |
| BBL/L vs. D1BL/L | -0.1333 | No | 0.7932 | 0.7932 | C-O |  |  |  |  |  |
| BBD/L vs. D1BD/L | 0.6278 | No | 0.1105 | 0.0904 | E-Q |  |  |  |  |  |
| BBB/L vs. RBB/L | -2.028 | **Yes** | **0.0134** | **0.0049** | A-Y |  |  |  |  |  |
| BAB/L vs. D1AB/L | -0.6556 | No | 0.2274 | 0.2067 | G-S |  |  |  |  |  |
| BAL/L vs. D1AL/L | -1.744 | No | 0.0813 | 0.0517 | I-U |  |  |  |  |  |
| BAD/L vs. D1AD/L | 2.106 | **Yes** | **0.0034** | **0.0009** | K-W |  |  |  |  |  |
| BAB/L vs. RAB/L | -1.628 | **Yes** | **0.0300** | **0.0148** | G-AA |  |  |  |  |  |
| BBB/D vs. BBL/D | -1.567 | No | 0.0888 | 0.0355 | B-D |  |  |  |  |  |
| BBB/D vs. BBD/D | -1.783 | No | 0.0760 | 0.0228 | B-F |  |  |  |  |  |
| BBL/D vs. BBD/D | -0.2167 | No | 0.6483 | 0.4538 | D-F |  |  |  |  |  |
| BBB/D vs. D1BB/D | -1.517 | **Yes** | **0.0190** | **0.0019** | B-N |  |  |  |  |  |
| BBL/D vs. D1BL/D | 0.5917 | No | 0.4300 | 0.2580 | D-P |  |  |  |  |  |
| BBD/D vs. D1BD/D | -0.2833 | No | 0.7247 | 0.7101 | F-R |  |  |  |  |  |
| BBB/D vs. RBB/D | 0.3250 | No | 0.7247 | 0.6564 | B-Z |  |  |  |  |  |
| BAB/D vs. D1AB/D | -0.9500 | No | 0.0560 | 0.0112 | H-T |  |  |  |  |  |
| BAL/D vs. D1AL/D | 0.1583 | No | 0.7247 | 0.7247 | J-V |  |  |  |  |  |
| BAD/D vs. D1AD/D | -0.9000 | No | 0.2369 | 0.1184 | L-X |  |  |  |  |  |
| BAB/D vs. RAB/D | -1.567 | No | 0.0888 | 0.0355 | H-AB |  |  |  |  |  |
| Test details | Mean 1 | Mean 2 | Mean diff, | SE of diff, | n1 | n2 | | t | | DF |
| BBB/L vs. BBL/L | 5.117 | 6.256 | -1.139 | 0.5958 | 18 | 18 | | 1.912 | | 17 |
| BBB/L vs. BBD/L | 5.117 | 7.150 | -2.033 | 0.4791 | 18 | 18 | | 4.244 | | 17 |
| BBL/L vs. BBD/L | 6.256 | 7.150 | -0.8944 | 0.3358 | 18 | 18 | | 2.663 | | 17 |
| BBB/L vs. D1BB/L | 5.117 | 7.517 | -2.400 | 0.5691 | 18 | 18 | | 4.217 | | 17 |
| BBL/L vs. D1BL/L | 6.256 | 6.389 | -0.1333 | 0.5007 | 18 | 18 | | 0.2663 | | 17 |
| BBD/L vs. D1BD/L | 7.150 | 6.522 | 0.6278 | 0.3497 | 18 | 18 | | 1.795 | | 17 |
| BBB/L vs. RBB/L | 5.117 | 7.144 | -2.028 | 0.6269 | 18 | 18 | | 3.234 | | 17 |
| BAB/L vs. D1AB/L | 1.178 | 1.833 | -0.6556 | 0.4994 | 18 | 18 | | 1.313 | | 17 |
| BAL/L vs. D1AL/L | 1.572 | 3.317 | -1.744 | 0.8338 | 18 | 18 | | 2.092 | | 17 |
| BAD/L vs. D1AD/L | 3.278 | 1.172 | 2.106 | 0.5259 | 18 | 18 | | 4.004 | | 17 |
| BAB/L vs. RAB/L | 1.178 | 2.806 | -1.628 | 0.6002 | 18 | 18 | | 2.712 | | 17 |
| BBB/D vs. BBL/D | 5.825 | 7.392 | -1.567 | 0.6540 | 12 | 12 | | 2.395 | | 11 |
| BBB/D vs. BBD/D | 5.825 | 7.608 | -1.783 | 0.6743 | 12 | 12 | | 2.645 | | 11 |
| BBL/D vs. BBD/D | 7.392 | 7.608 | -0.2167 | 0.2790 | 12 | 12 | | 0.7765 | | 11 |
| BBB/D vs. D1BB/D | 5.825 | 7.342 | -1.517 | 0.3741 | 12 | 12 | | 4.054 | | 11 |
| BBL/D vs. D1BL/D | 7.392 | 6.800 | 0.5917 | 0.4960 | 12 | 12 | | 1.193 | | 11 |
| BBD/D vs. D1BD/D | 5.825 | 6.108 | -0.2833 | 0.7427 | 12 | 12 | | 0.3815 | | 11 |
| BBB/D vs. RBB/D | 2.383 | 2.058 | 0.3250 | 0.7109 | 12 | 12 | | 0.4572 | | 11 |
| BAB/D vs. D1AB/D | 1.317 | 2.267 | -0.9500 | 0.3122 | 12 | 12 | | 3.042 | | 11 |
| BAL/D vs. D1AL/D | 1.958 | 1.800 | 0.1583 | 0.4382 | 12 | 12 | | 0.3613 | | 11 |
| BAD/D vs. D1AD/D | 2.383 | 3.283 | -0.9000 | 0.5314 | 12 | 12 | | 1.694 | | 11 |
| BAB/D vs. RAB/D | 5.825 | 7.392 | -1.567 | 0.6540 | 12 | 12 | | 2.395 | | 11 |

| **Uncorrected Fisher's LSD** | Mean diff, | 95.00% CI of diff, | Below threshold? | Summary | Individual P Value |  |
| --- | --- | --- | --- | --- | --- | --- |
| BBB/L vs. BBL/L | -1.139 | -2.396 to 0.1181 | No | ns | 0.0729 |  |
| BBB/L vs. BBD/L | -2.033 | -3.044 to -1.023 | Yes | *** | 0.0005 |  |
| BBL/L vs. BBD/L | -0.8944 | -1.603 to -0.1859 | Yes | * | 0.0164 |  |
| BBB/L vs. D1BB/L | -2.400 | -3.601 to -1.199 | Yes | *** | 0.0006 |  |
| BBL/L vs. D1BL/L | -0.1333 | -1.190 to 0.9231 | No | ns | 0.7932 |  |
| BBD/L vs. D1BD/L | 0.6278 | -0.1100 to 1.366 | No | ns | 0.0904 |  |
| BBB/L vs. RBB/L | -2.028 | -3.351 to -0.7050 | Yes | ** | 0.0049 |  |
| BAB/L vs. D1AB/L | -0.6556 | -1.709 to 0.3980 | No | ns | 0.2067 |  |
| BAL/L vs. D1AL/L | -1.744 | -3.504 to 0.01475 | No | ns | 0.0517 |  |
| BAD/L vs. D1AD/L | 2.106 | 0.9960 to 3.215 | Yes | *** | 0.0009 |  |
| BAB/L vs. RAB/L | -1.628 | -2.894 to -0.3616 | Yes | * | 0.0148 |  |

| BBB/D vs. BBL/D | -1.567 | -3.006 to -0.1271 | Yes | * | 0.0355 |
| --- | --- | --- | --- | --- | --- |
| BBB/D vs. BBD/D | -1.783 | -3.267 to -0.2992 | Yes | * | 0.0228 |
| BBL/D vs. BBD/D | -0.2167 | -0.8308 to 0.3975 | No | ns | 0.4538 |
| BBB/D vs. D1BB/D | -1.517 | -2.340 to -0.6932 | Yes | ** | 0.0019 |
| BBL/D vs. D1BL/D | 0.5917 | -0.4999 to 1.683 | No | ns | 0.2580 |
| BBD/D vs. D1BD/D | -0.2833 | -1.918 to 1.351 | No | ns | 0.7101 |
| BBB/D vs. RBB/D | 0.3250 | -1.240 to 1.890 | No | ns | 0.6564 |
| BAB/D vs. D1AB/D | -0.9500 | -1.637 to -0.2627 | Yes | * | 0.0112 |
| BAL/D vs. D1AL/D | 0.1583 | -0.8062 to 1.123 | No | ns | 0.7247 |
| BAD/D vs. D1AD/D | -0.9000 | -2.070 to 0.2697 | No | ns | 0.1184 |

**SWEET**

Sweet taste preference differed significantly across time points in both sessions:

SD/L: (F(5.948, 101.1) = 5.349, p < 0.0001, R² = 0.2393)

SD/D: (F(4.195, 46.14) = 2.925, p = 0.0289, R² = 0.2100)

| Number of families | 1 |  |  |  |  |  |  | |  |
| --- | --- | --- | --- | --- | --- | --- | --- | --- | --- |
| Number of comparisons per family | 22 |  |  |  |  |  |  | |  |
| Q | 0.1 |  |  |  |  |  |  | |  |
|  |  |  |  |  |  |  |  | |  |
| **Original FDR method of**  **Benjamini and Hochberg** | Mean Diff. | Discovery? | q value | Individual P Value |  |  |  |  |  |
| BBB/L vs. BBL/L | -1.175 | No | 0.5782 | 0.3942 | A-C |  |  |  |  |
| BBB/L vs. BBD/L | -0.75 | No | 0.7103 | 0.591 | A-E |  |  |  |  |
| BBL/L vs. BBD/L | 0.425 | No | 0.7103 | 0.6134 | C-E |  |  |  |  |
| BBB/L vs. D1BB/L | 1.733 | No | 0.5298 | 0.1096 | A-M |  |  |  |  |
| BBL/L vs. D1BL/L | 0.55 | No | 0.6607 | 0.5105 | C-O |  |  |  |  |
| BBD/L vs. D1BD/L | -1.008 | No | 0.5298 | 0.2378 | E-Q |  |  |  |  |
| BBB/L vs. RBB/L | 1.408 | No | 0.5298 | 0.0918 | A-Y |  |  |  |  |
| BAB/L vs. D1AB/L | -0.6333 | No | 0.5298 | 0.2342 | G-S |  |  |  |  |
| BAL/L vs. D1AL/L | -0.025 | No | 0.9866 | 0.9624 | I-U |  |  |  |  |
| BAD/L vs. D1AD/L | 0.325 | No | 0.7861 | 0.7146 | K-W |  |  |  |  |
| BAB/L vs. RAB/L | -1.433 | No | 0.5298 | 0.1827 | G-AA |  |  |  |  |
| BBB/D vs. BBL/D | -1.725 | No | 0.5298 | 0.2408 | B-D |  |  |  |  |
| BBB/D vs. BBD/D | -1.708 | No | 0.5298 | 0.1432 | B-F |  |  |  |  |
| BBL/D vs. BBD/D | 0.01667 | No | 0.9866 | 0.9866 | D-F |  |  |  |  |
| BBB/D vs. D1BB/D | -0.9083 | No | 0.5927 | 0.431 | B-N |  |  |  |  |
| BBL/D vs. D1BL/D | -1.167 | No | 0.532 | 0.3059 | D-P |  |  |  |  |
| BBD/D vs. D1BD/D | -0.95 | No | 0.532 | 0.3386 | F-R |  |  |  |  |
| BBB/D vs. RBB/D | -1.225 | No | 0.532 | 0.3337 | B-Z |  |  |  |  |
| BAB/D vs. D1AB/D | -1.508 | No | 0.5298 | 0.1503 | H-T |  |  |  |  |
| BAL/D vs. D1AL/D | -1.508 | No | 0.5298 | 0.172 | J-V |  |  |  |  |
| BAD/D vs. D1AD/D | -2.733 | No | 0.2015 | 0.0092 | L-X |  |  |  |  |
| BAB/D vs. RAB/D | -1.233 | No | 0.532 | 0.2854 | H-AB |  |  |  |  |
| Test details | Mean 1 | Mean 2 | Mean Diff. | SE of diff. | n1 | n2 | t | | DF |
| BBB/L vs. BBL/L | 3.883 | 5.058 | -1.175 | 1.325 | 12 | 12 | 0.8867 | | 11 |
| BBB/L vs. BBD/L | 3.883 | 4.633 | -0.75 | 1.355 | 12 | 12 | 0.5535 | | 11 |
| BBL/L vs. BBD/L | 5.058 | 4.633 | 0.425 | 0.8174 | 12 | 12 | 0.5199 | | 11 |
| BBB/L vs. D1BB/L | 3.883 | 2.15 | 1.733 | 0.9958 | 12 | 12 | 1.741 | | 11 |
| BBL/L vs. D1BL/L | 5.058 | 4.508 | 0.55 | 0.8087 | 12 | 12 | 0.6801 | | 11 |
| BBD/L vs. D1BD/L | 4.633 | 5.642 | -1.008 | 0.8078 | 12 | 12 | 1.248 | | 11 |
| BBB/L vs. RBB/L | 3.883 | 2.475 | 1.408 | 0.7625 | 12 | 12 | 1.847 | | 11 |
| BAB/L vs. D1AB/L | 4.717 | 5.35 | -0.6333 | 0.5032 | 12 | 12 | 1.259 | | 11 |
| BAL/L vs. D1AL/L | 5.475 | 5.5 | -0.025 | 0.5189 | 12 | 12 | 0.0482 | | 11 |
| BAD/L vs. D1AD/L | 4.958 | 4.633 | 0.325 | 0.866 | 12 | 12 | 0.3753 | | 11 |
| BAB/L vs. RAB/L | 4.717 | 6.15 | -1.433 | 1.008 | 12 | 12 | 1.422 | | 11 |
| BBB/D vs. BBL/D | 3.958 | 5.683 | -1.725 | 1.391 | 12 | 12 | 1.24 | | 11 |
| BBB/D vs. BBD/D | 3.958 | 5.667 | -1.708 | 1.084 | 12 | 12 | 1.576 | | 11 |
| BBL/D vs. BBD/D | 5.683 | 5.667 | 0.01667 | 0.9671 | 12 | 12 | 0.0172 | | 11 |
| BBB/D vs. D1BB/D | 3.958 | 4.867 | -0.9083 | 1.111 | 12 | 12 | 0.8174 | | 11 |
| BBL/D vs. D1BL/D | 5.683 | 6.85 | -1.167 | 1.086 | 12 | 12 | 1.074 | | 11 |
| BBD/D vs. D1BD/D | 5.667 | 6.617 | -0.95 | 0.9495 | 12 | 12 | 1.001 | | 11 |
| BBB/D vs. RBB/D | 3.958 | 5.183 | -1.225 | 1.211 | 12 | 12 | 1.011 | | 11 |
| BAB/D vs. D1AB/D | 5.758 | 7.267 | -1.508 | 0.9756 | 12 | 12 | 1.546 | | 11 |
| BAL/D vs. D1AL/D | 5.275 | 6.783 | -1.508 | 1.033 | 12 | 12 | 1.461 | | 11 |
| BAD/D vs. D1AD/D | 5.625 | 8.358 | -2.733 | 0.8663 | 12 | 12 | 3.155 | | 11 |
| BAB/D vs. RAB/D | 5.758 | 6.992 | -1.233 | 1.098 | 12 | 12 | 1.123 | | 11 |

**SALTY**

Salt taste preference differed significantly across time points in both sessions:

SD/L: (F(6.530, 111.0) = 10.79, p < 0.0001, R² = 0.3883)

SD/D: (F(5.151, 56.66) = 4.373, p = 0.0018, R² = 0.2844)

| Number of families | 1 | |  |  |  |  | |  | |  |  |
| --- | --- | --- | --- | --- | --- | --- | --- | --- | --- | --- | --- |
| Number of comparisons per family | 22 | |  |  |  |  | |  | |  |  |
| Q | 0.1 | |  |  |  |  | |  | |  |  |
|  |  | |  |  |  |  | |  | |  |  |
| **Original FDR method of Benjamini and Hochberg** | | Mean Diff | Discovery? | q value | Individual P Value |  |  | |  |  |  |
| BBB/L vs. BBL/L | | -0.5083 | No | 0.6197 | 0.4983 | A-C | |  |  |  |  |
| BBB/L vs. BBD/L | | 0.8167 | No | 0.6197 | 0.4528 | A-E | |  |  |  |  |
| BBL/L vs. BBD/L | | 1.325 | No | 0.5176 | 0.3097 | C-E | |  |  |  |  |
| BBB/L vs. D1BB/L | | 1.217 | No | 0.2238 | 0.0814 | A-M | |  |  |  |  |
| BBL/L vs. D1BL/L | | 2.083 | No | 0.1191 | 0.0297 | C-O | |  |  |  |  |
| BBD/L vs. D1BD/L | | 0.3167 | No | 0.8005 | 0.7277 | E-Q | |  |  |  |  |
| BBB/L vs. RBB/L | | -0.1083 | No | 0.9099 | 0.9099 | A-Y | |  |  |  |  |
| BAB/L vs. D1AB/L | 0.7583 | | No | 0.4965 | 0.2483 | G-S | |  |  |  |  |
| BAL/L vs. D1AL/L | -0.4167 | | No | 0.6197 | 0.507 | I-U | |  |  |  |  |
| BAD/L vs. D1AD/L | -1.908 | | No | 0.1191 | 0.0148 | K-W | |  |  |  |  |
| BAB/L vs. RAB/L | 2.708 | | No | 0.1191 | 0.0318 | G-AA | |  |  |  |  |
| BBB/D vs. BBL/D | 1.592 | | No | 0.3983 | 0.181 | B-D | |  |  |  |  |
| BBB/D vs. BBD/D | 2.675 | | No | 0.1191 | 0.0149 | B-F | |  |  |  |  |
| BBL/D vs. BBD/D | 1.083 | | No | 0.4979 | 0.2716 | D-F | |  |  |  |  |
| BBB/D vs. D1BB/D | 2.717 | | No | 0.1191 | 0.0325 | B-N | |  |  |  |  |
| BBL/D vs. D1BL/D | 1.85 | | No | 0.3135 | 0.1283 | D-P | |  |  |  |  |
| BBD/D vs. D1BD/D | 0.2083 | | No | 0.8828 | 0.8427 | F-R | |  |  |  |  |
| BBB/D vs. RBB/D | 2.05 | | No | 0.2238 | 0.0762 | B-Z | |  |  |  |  |
| BAB/D vs. D1AB/D | -1.025 | | No | 0.5176 | 0.3365 | H-T | |  |  |  |  |
| BAL/D vs. D1AL/D | 2.225 | | No | 0.1191 | 0.0288 | J-V | |  |  |  |  |
| BAD/D vs. D1AD/D | 0.925 | | No | 0.5176 | 0.3529 | L-X | |  |  |  |  |
| BAB/D vs. RAB/D | 0.5583 | | No | 0.7214 | 0.623 | H-AB | |  |  |  |  |
| Test details | Mean 1 | | Mean 2 | Mean Diff. | SE of diff. | n1 | | n2 | | t | DF |
| BBB/L vs. BBL/L | 4 | | 4.508 | -0.5083 | 0.726 | 12 | | 12 | | 0.7002 | 11 |
| BBB/L vs. BBD/L | 4 | | 3.183 | 0.8167 | 1.049 | 12 | | 12 | | 0.7784 | 11 |
| BBL/L vs. BBD/L | 4.508 | | 3.183 | 1.325 | 1.244 | 12 | | 12 | | 1.065 | 11 |
| BBB/L vs. D1BB/L | 4 | | 2.783 | 1.217 | 0.6342 | 12 | | 12 | | 1.918 | 11 |
| BBL/L vs. D1BL/L | 4.508 | | 2.425 | 2.083 | 0.8344 | 12 | | 12 | | 2.497 | 11 |
| BBD/L vs. D1BD/L | 3.183 | | 2.867 | 0.3167 | 0.8865 | 12 | | 12 | | 0.3572 | 11 |
| BBB/L vs. RBB/L | 4 | | 4.108 | -0.1083 | 0.9351 | 12 | | 12 | | 0.1158 | 11 |
| BAB/L vs. D1AB/L | 7.217 | | 6.458 | 0.7583 | 0.622 | 12 | | 12 | | 1.219 | 11 |
| BAL/L vs. D1AL/L | 6.958 | | 7.375 | -0.4167 | 0.6075 | 12 | | 12 | | 0.6859 | 11 |
| BAD/L vs. D1AD/L | 4.508 | | 6.417 | -1.908 | 0.661 | 12 | | 12 | | 2.887 | 11 |
| BAB/L vs. RAB/L | 7.217 | | 4.508 | 2.708 | 1.102 | 12 | | 12 | | 2.459 | 11 |
| BBB/D vs. BBL/D | 5.825 | | 4.233 | 1.592 | 1.115 | 12 | | 12 | | 1.428 | 11 |
| BBB/D vs. BBD/D | 5.825 | | 3.15 | 2.675 | 0.9281 | 12 | | 12 | | 2.882 | 11 |
| BBL/D vs. BBD/D | 4.233 | | 3.15 | 1.083 | 0.9359 | 12 | | 12 | | 1.158 | 11 |
| BBB/D vs. D1BB/D | 5.825 | | 3.108 | 2.717 | 1.111 | 12 | | 12 | | 2.446 | 11 |
| BBL/D vs. D1BL/D | 4.233 | | 2.383 | 1.85 | 1.125 | 12 | | 12 | | 1.645 | 11 |
| BBD/D vs. D1BD/D | 3.15 | | 2.942 | 0.2083 | 1.025 | 12 | | 12 | | 0.2032 | 11 |
| BBB/D vs. RBB/D | 5.825 | | 3.775 | 2.05 | 1.048 | 12 | | 12 | | 1.957 | 11 |
| BAB/D vs. D1AB/D | 4.383 | | 5.408 | -1.025 | 1.02 | 12 | | 12 | | 1.005 | 11 |
| BAL/D vs. D1AL/D | 6.725 | | 4.5 | 2.225 | 0.8852 | 12 | | 12 | | 2.514 | 11 |
| BAD/D vs. D1AD/D | 7.075 | | 6.15 | 0.925 | 0.9537 | 12 | | 12 | | 0.9699 | 11 |
| BAB/D vs. RAB/D | 4.383 | | 3.825 | 0.5583 | 1.104 | 12 | | 12 | | 0.5057 | 11 |

**PICANT**

Spicy taste preference differed significantly across time points in both sessions:

SD/L: (F(5.747, 97.69) = 8.369, p < 0.0001, R² = 0.3299)

SD/D: (F(4.615, 50.76) = 4.255, p = 0.0033, R² = 0.2789)

| Number of families | | 1 | |  | |  | |  | |  | |  | | | | |  |  |
| --- | --- | --- | --- | --- | --- | --- | --- | --- | --- | --- | --- | --- | --- | --- | --- | --- | --- | --- |
| Number of comparisons per family | | 22 | |  | |  | |  | |  | |  | | | | |  |  |
| Q | | 0.1 | |  | |  | |  | |  | |  | | | | |  |  |
| **Original FDR method of Benjamini and Hochberg** | | Mean Diff. | | Discovery? | | q value | | Individual P Value | |  |  | |  | | |  |  |  |
| BBB/L vs. BBL/L | | 2.208 | | No | | 0.363 | | 0.0908 | | A-C | |  |  |  |  |  |  |  |
| BBB/L vs. BBD/L | | 2.9 | | **Yes** | | **0.0554** | | **0.005** | | A-E | |  |  |  |  |  |  |  |
| BBL/L vs. BBD/L | | 0.6917 | | No | | 0.6903 | | 0.4291 | | C-E | |  |  |  |  |  |  |  |
| BBB/L vs. D1BB/L | | -0.225 | | No | | 0.7937 | | 0.7215 | | A-M | |  |  |  |  |  |  |  |
| BBL/L vs. D1BL/L | | 0.775 | | No | | 0.5301 | | 0.265 | | C-O | |  |  |  |  |  |  |  |
| BBD/L vs. D1BD/L | | -0.3833 | | No | | 0.7509 | | 0.6485 | | E-Q | |  |  |  |  |  |  |  |
| BBB/L vs. RBB/L | | -0.5083 | | No | | 0.6903 | | 0.4424 | | A-Y | |  |  |  |  |  |  |  |
| BAB/L vs. D1AB/L | | 0.2333 | | No | | 0.6935 | | 0.5044 | | G-S | |  |  |  |  |  |  |  |
| BAL/L vs. D1AL/L | | -0.3083 | | No | | 0.5808 | | 0.3168 | | I-U | |  |  |  |  |  |  |  |
| BAD/L vs. D1AD/L | | -1.175 | | No | | 0.363 | | 0.132 | | K-W | |  |  |  |  |  |  |  |
| BAB/L vs. RAB/L | | 0.5333 | | No | | 0.4778 | | 0.2172 | | G-AA | |  |  |  |  |  |  |  |
| BBB/D vs. BBL/D | | 2.192 | | No | | 0.363 | | 0.1174 | | B-D | |  |  |  |  |  |  |  |
| BBB/D vs. BBD/D | | 2.108 | | No | | 0.2406 | | 0.0328 | | B-F | |  |  |  |  |  |  |  |
| BBL/D vs. BBD/D | | -0.08333 | | No | | 0.9317 | | 0.9317 | | D-F | |  |  |  |  |  |  |  |
| BBB/D vs. D1BB/D | | 0.5833 | | No | | 0.7509 | | 0.6244 | | B-N | |  |  |  |  |  |  |  |
| BBL/D vs. D1BL/D | | 0.8583 | | No | | 0.6903 | | 0.4706 | | D-P | |  |  |  |  |  |  |  |
| BBD/D vs. D1BD/D | | 1.692 | | No | | 0.366 | | 0.1497 | | F-R | |  |  |  |  |  |  |  |
| BBB/D vs. RBB/D | | 3.433 | | **Yes** | | **0.0554** | | **0.0045** | | B-Z | |  |  |  |  |  |  |  |
| BAB/D vs. D1AB/D | | -0.1333 | | No | | 0.9317 | | 0.892 | | H-T | |  |  |  |  |  |  |  |
| BAL/D vs. D1AL/D | | 1.858 | | No | | 0.363 | | 0.1243 | | J-V | |  |  |  |  |  |  |  |
| BAD/D vs. D1AD/D | | 0.475 | | No | | 0.7509 | | 0.5831 | | L-X | |  |  |  |  |  |  |  |
| BAB/D vs. RAB/D | | 2.308 | | No | | 0.2965 | | 0.0539 | | H-AB | |  |  |  |  |  |  |  |
| Test details | | Mean 1 | | Mean 2 | | Mean Diff. | | SE of diff. | | n1 | | n2 | | | | | t | DF |
| BBB/L vs. BBL/L | | 6.975 | | 4.767 | | 2.208 | | 1.192 | | 12 | | 12 | | | | | 1.853 | 11 |
| BBB/L vs. BBD/L | | 6.975 | | 4.075 | | 2.9 | | 0.8304 | | 12 | | 12 | | | | | 3.492 | 11 |
| BBL/L vs. BBD/L | | 4.767 | | 4.075 | | 0.6917 | | 0.8425 | | 12 | | 12 | | | | | 0.821 | 11 |
| BBB/L vs. D1BB/L | | 6.975 | | 7.2 | | -0.225 | | 0.6152 | | 12 | | 12 | | | | | 0.3657 | 11 |
| BBL/L vs. D1BL/L | | 4.767 | | 3.992 | | 0.775 | | 0.6599 | | 12 | | 12 | | | | | 1.174 | 11 |
| BBD/L vs. D1BD/L | | 4.075 | | 4.458 | | -0.3833 | | 0.8181 | | 12 | | 12 | | | | | 0.4686 | 11 |
| BBB/L vs. RBB/L | | 6.975 | | 7.483 | | -0.5083 | | 0.638 | | 12 | | 12 | | | | | 0.7968 | 11 |
| BAB/L vs. D1AB/L | | 7.733 | | 7.5 | | 0.2333 | | 0.3381 | | 12 | | 12 | | | | | 0.6902 | 11 |
| BAL/L vs. D1AL/L | | 7.283 | | 7.592 | | -0.3083 | | 0.294 | | 12 | | 12 | | | | | 1.049 | 11 |
| BAD/L vs. D1AD/L | | 5.667 | | 6.842 | | -1.175 | | 0.7222 | | 12 | | 12 | | | | | 1.627 | 11 |
| BAB/L vs. RAB/L | | 7.733 | | 7.2 | | 0.5333 | | 0.4074 | | 12 | | 12 | | | | | 1.309 | 11 |
| BBB/D vs. BBL/D | | 7.208 | | 5.017 | | 2.192 | | 1.29 | | 12 | | 12 | | | | | 1.699 | 11 |
| BBB/D vs. BBD/D | | 7.208 | | 5.1 | | 2.108 | | 0.864 | | 12 | | 12 | | | | | 2.44 | 11 |
| BBL/D vs. BBD/D | | 5.017 | | 5.1 | | -0.08333 | | 0.9503 | | 12 | | 12 | | | | | 0.08769 | 11 |
| BBB/D vs. D1BB/D | | 7.208 | | 6.625 | | 0.5833 | | 1.158 | | 12 | | 12 | | | | | 0.5037 | 11 |
| BBL/D vs. D1BL/D | | 5.017 | | 4.158 | | 0.8583 | | 1.149 | | 12 | | 12 | | | | | 0.7471 | 11 |
| BBD/D vs. D1BD/D | | 5.1 | | 3.408 | | 1.692 | | 1.092 | | 12 | | 12 | | | | | 1.549 | 11 |
| BBB/D vs. RBB/D | | 7.208 | | 3.775 | | 3.433 | | 0.9651 | | 12 | | 12 | | | | | 3.557 | 11 |
| BAB/D vs. D1AB/D | | 6.133 | | 6.267 | | -0.1333 | | 0.9592 | | 12 | | 12 | | | | | 0.139 | 11 |
| BAL/D vs. D1AL/D | | 7.775 | | 5.917 | | 1.858 | | 1.117 | | 12 | | 12 | | | | | 1.664 | 11 |
| BAD/D vs. D1AD/D | | 7.442 | | 6.967 | | 0.475 | | 0.84 | | 12 | | 12 | | | | | 0.5655 | 11 |
| BAB/D vs. RAB/D | | 6.133 | | 3.825 | | 2.308 | | 1.07 | | 12 | | 12 | | | | | 2.158 | 11 |
| **Uncorrected Fisher's LSD** | Mean diff, | | 95.00% CI of diff, | | Below threshold? | | Summary | | Individual P Value | | | | |  |  |  |  |  |
| BBB/L vs. BBD/L | 2.367 | | 0.4194 to 4.314 | | Yes | | * | | 0.0201 | | | | |  |  |  |  |  |
| BBB/D vs. RBB/D | 3.433 | | 1.309 to 5.557 | | Yes | | ** | | 0.0045 | | | | |  |  |  |  |  |

**GREASY**

Greasy taste preference differed significantly across time points in both sessions:

SD/L: (F(6.006, 102.1) = 7.787, p < 0.0001, R² = 0.3141)

SD/D: (F(4.209, 46.30) = 5.275, p = 0.0012, R² = 0.3241)

| Number of families | 1 |  |  |  | |  | |  |  | |  | |  | |
| --- | --- | --- | --- | --- | --- | --- | --- | --- | --- | --- | --- | --- | --- | --- |
| Number of comparisons per family | 22 |  |  |  | |  | |  |  | |  | |  | |
| Q | 0.1 |  |  |  | |  | |  |  | |  | |  | |
|  |  |  |  |  | |  | |  |  | |  | |  | |
| **Original FDR method of**  **Benjamini and Hochberg** | Mean Diff. | Discovery? | q value |  | Individual  P Value | | | |  |  | |  | |  |
| BBB/L vs. BBL/L | 0.5417 | No | 0.754 | 0.5141 | |  | A-C | |  | |  |  |  |  |
| BBB/L vs. BBD/L | 1.892 | No | 0.4146 | 0.081 | |  | A-E | |  | |  |  |  |  |
| BBL/L vs. BBD/L | 1.35 | No | 0.4146 | 0.0809 | |  | C-E | |  | |  |  |  |  |
| BBB/L vs. D1BB/L | 0.825 | No | 0.7227 | 0.427 | |  | A-M | |  | |  |  |  |  |
| BBL/L vs. D1BL/L | 0.5417 | No | 0.6833 | 0.3727 | |  | C-O | |  | |  |  |  |  |
| BBD/L vs. D1BD/L | -0.7833 | No | 0.4146 | 0.1009 | |  | E-Q | |  | |  |  |  |  |
| BBB/L vs. RBB/L | 0.2667 | No | 0.8768 | 0.7572 | |  | A-Y | |  | |  |  |  |  |
| BAB/L vs. D1AB/L | 0.325 | No | 0.754 | 0.5074 | |  | G-S | |  | |  |  |  |  |
| BAL/L vs. D1AL/L | -0.4667 | No | 0.5002 | 0.2011 | |  | I-U | |  | |  |  |  |  |
| BAD/L vs. D1AD/L | -1.417 | No | 0.4931 | 0.1569 | |  | K-W | |  | |  |  |  |  |
| BAB/L vs. RAB/L | 1.058 | No | 0.5986 | 0.2721 | |  | G-AA | | | |  |  |  |  |
| BBB/D vs. BBL/D | 1.925 | No | 0.4146 | 0.1131 | |  | B-D | |  | |  |  |  |  |
| BBB/D vs. BBD/D | 1.817 | No | 0.3707 | 0.0337 | |  | B-F | |  | |  |  |  |  |
| BBL/D vs. BBD/D | -0.1083 | No | 0.9232 | 0.8812 | |  | D-F | |  | |  |  |  |  |
| BBB/D vs. D1BB/D | 1.183 | No | 0.6833 | 0.3644 | |  | B-N | |  | |  |  |  |  |
| BBL/D vs. D1BL/D | 0.4083 | No | 0.8238 | 0.5991 | |  | D-P | |  | |  |  |  |  |
| BBD/D vs. D1BD/D | -0.0083 | No | 0.9903 | 0.9903 | |  | F-R | |  | |  |  |  |  |
| BBB/D vs. RBB/D | 0.3 | No | 0.8768 | 0.7338 | |  | B-Z | |  | |  |  |  |  |
| BAB/D vs. D1AB/D | -2.233 | **Yes** | **0.0271** | **0.0012** | |  | H-T | |  | |  |  |  |  |
| BAL/D vs. D1AL/D | -0.2083 | No | 0.9232 | 0.8439 | |  | J-V | |  | |  |  |  |  |
| BAD/D vs. D1AD/D | -0.3833 | No | 0.8466 | 0.6542 | |  | L-X | |  | |  |  |  |  |
| BAB/D vs. RAB/D | -0.8667 | No | 0.5002 | 0.2046 | |  | H-AB | | | |  |  |  |  |
| Test details | Mean 1 | Mean 2 | Mean Diff. | SE of diff. | |  | | n1 | n2 | | t | | DF | |
| BBB/L vs. BBL/L | 5.908 | 5.367 | 0.5417 | 0.8034 | |  | | 12 | 12 | | 0.6742 | | 11 | |
| BBB/L vs. BBD/L | 5.908 | 4.017 | 1.892 | 0.9847 | |  | | 12 | 12 | | 1.921 | | 11 | |
| BBL/L vs. BBD/L | 5.367 | 4.017 | 1.35 | 0.7023 | |  | | 12 | 12 | | 1.922 | | 11 | |
| BBB/L vs. D1BB/L | 5.908 | 5.083 | 0.825 | 1 | |  | | 12 | 12 | | 0.8247 | | 11 | |
| BBL/L vs. D1BL/L | 5.367 | 4.825 | 0.5417 | 0.5829 | |  | | 12 | 12 | | 0.9293 | | 11 | |
| BBD/L vs. D1BD/L | 4.017 | 4.8 | -0.7833 | 0.4374 | |  | | 12 | 12 | | 1.791 | | 11 | |
| BBB/L vs. RBB/L | 5.908 | 5.642 | 0.2667 | 0.8413 | |  | | 12 | 12 | | 0.317 | | 11 | |
| BAB/L vs. D1AB/L | 7.692 | 7.367 | 0.325 | 0.4744 | |  | | 12 | 12 | | 0.6851 | | 11 | |
| BAL/L vs. D1AL/L | 7.933 | 8.4 | -0.4667 | 0.3432 | |  | | 12 | 12 | | 1.36 | | 11 | |
| BAD/L vs. D1AD/L | 6.117 | 7.533 | -1.417 | 0.9325 | |  | | 12 | 12 | | 1.519 | | 11 | |
| BAB/L vs. RAB/L | 7.692 | 6.633 | 1.058 | 0.9153 | |  | | 12 | 12 | | 1.156 | | 11 | |
| BBB/D vs. BBL/D | 6.167 | 4.242 | 1.925 | 1.118 | |  | | 12 | 12 | | 1.722 | | 11 | |
| BBB/D vs. BBD/D | 6.167 | 4.35 | 1.817 | 0.7491 | |  | | 12 | 12 | | 2.425 | | 11 | |
| BBL/D vs. BBD/D | 4.242 | 4.35 | -0.1083 | 0.7082 | |  | | 12 | 12 | | 0.153 | | 11 | |
| BBB/D vs. D1BB/D | 6.167 | 4.983 | 1.183 | 1.251 | |  | | 12 | 12 | | 0.9461 | | 11 | |
| BBL/D vs. D1BL/D | 4.242 | 3.833 | 0.4083 | 0.7544 | |  | | 12 | 12 | | 0.5412 | | 11 | |
| BBD/D vs. D1BD/D | 4.35 | 4.358 | -0.008333 | 0.6709 | |  | | 12 | 12 | | 0.0124 | | 11 | |
| BBB/D vs. RBB/D | 6.167 | 5.867 | 0.3 | 0.86 | |  | | 12 | 12 | | 0.3489 | | 11 | |
| BAB/D vs. D1AB/D | 5.6 | 7.833 | -2.233 | 0.5179 | |  | | 12 | 12 | | 4.312 | | 11 | |
| BAL/D vs. D1AL/D | 7.342 | 7.55 | -0.2083 | 1.033 | |  | | 12 | 12 | | 0.2017 | | 11 | |
| BAD/D vs. D1AD/D | 7.408 | 7.792 | -0.3833 | 0.8326 | |  | | 12 | 12 | | 0.4604 | | 11 | |
| BAB/D vs. RAB/D | 5.6 | 6.467 | -0.8667 | 0.6427 | |  | | 12 | 12 | | 1.348 | | 11 | |

**Uncorrected Fisher's LSD** Mean diff, 90.00% CI of diff, Below threshold? Summary Individual P Value

| BAB/D vs. D1AB/D -2.233 | -3.163 to -1.303 | Yes | ** 0.0012 |
| --- | --- | --- | --- |

**Table 12: Statistics for Visual Analogue Scale Assessment for Satiety, and Sweet, Salty, Picant, and Greasy Taste Preferences** using RM ANOVA comparing the equal SD/L and SD/D groups of 12 subjects with post-hoc tests (Benjamini–Hochberg FDR and Uncorrected Fisher’s LSD when applicable).

**SATIETY**

Satiety differed significantly between SD/L and SD/D groups (F(6.741, 74.15) = 30.28, p < 0.0001, R² = 0.7335).

| Number of families | 1 |  |  |  |  | |  |  | |  | |
| --- | --- | --- | --- | --- | --- | --- | --- | --- | --- | --- | --- |
| Number of comparisons per family | 14 |  |  |  |  | |  |  | |  | |
| Q | 0.1 |  |  |  |  | |  |  | |  | |
| **Original FDR method of Benjamini and Hochberg** | Mean Diff. | Discovery? | q value | Individual P Value |  |  | | |  | |  |
| D1BB/L vs. D1BB/D | -0.2917 | No | 0.6956 | 0.6362 | M-N | |  |  | |  | |
| D1BL/L vs. D1BL/D | -0.8583 | No | 0.3839 | 0.1645 | O-P | |  |  | |  | |
| D1BD/L vs. D1BD/D | -0.3083 | No | 0.6956 | 0.6171 | Q-R | |  |  | |  |  |
| RBB/L vs. RBB/D | 0.8667 | No | 0.3839 | 0.1605 | Y-Z | |  |  | |  |  |
| D1AB/L vs. D1AB/D | -0.06667 | No | 0.9139 | 0.9139 | S-T | |  |  | |  |  |
| D1AL/L vs. D1AL/D | -0.9333 | No | 0.3839 | 0.1308 | U-V | |  |  | |  |  |
| D1AD/L vs. D1AD/D | -0.2833 | No | 0.6956 | 0.6459 | W-X | |  |  | |  |  |
| RAB/L vs. RAB/D | -0.3 | No | 0.6956 | 0.6266 | AA-AB | | | | |  |  |
| BBB/L vs. BBB/D | -0.8 | No | 0.3901 | 0.1951 | A-B | |  |  | |  |  |
| BBL/L vs. BBL/D | -1.375 | No | 0.123 | 0.0264 | C-D | |  |  | |  |  |
| BBD/L vs. BBD/D | -0.6083 | No | 0.5673 | 0.3242 | E-F | |  |  | |  |  |
| BAB/L vs. BAB/D | -1.425 | No | 0.123 | 0.0214 | G-H | |  |  | |  |  |
| BAL/L vs. BAL/D | 0.5417 | No | 0.591 | 0.3799 | I-J | |  |  | |  |  |
| BAD/L vs. BAD/D | 1.683 | Yes | 0.0932 | 0.0067 | K-L | |  |  | |  |  |
| Test details | Mean 1 | Mean 2 | Mean Diff. | SE of diff. | n1 | | n2 | t | | DF | |
| D1BB/L vs. D1BB/D | 7.05 | 7.342 | -0.2917 | 0.616 | 12 | | 12 | 0.4735 | | 297 | |
| D1BL/L vs. D1BL/D | 5.942 | 6.8 | -0.8583 | 0.616 | 12 | | 12 | 1.393 | | 297 | |
| D1BD/L vs. D1BD/D | 6.342 | 6.65 | -0.3083 | 0.616 | 12 | | 12 | 0.5005 | | 297 | |
| RBB/L vs. RBB/D | 6.975 | 6.108 | 0.8667 | 0.616 | 12 | | 12 | 1.407 | | 297 | |
| D1AB/L vs. D1AB/D | 1.992 | 2.058 | -0.06667 | 0.616 | 12 | | 12 | 0.1082 | | 297 | |
| D1AL/L vs. D1AL/D | 1.333 | 2.267 | -0.9333 | 0.616 | 12 | | 12 | 1.515 | | 297 | |
| D1AD/L vs. D1AD/D | 1.517 | 1.8 | -0.2833 | 0.616 | 12 | | 12 | 0.46 | | 297 | |
| RAB/L vs. RAB/D | 2.983 | 3.283 | -0.3 | 0.616 | 12 | | 12 | 0.487 | | 297 | |
| BBB/L vs. BBB/D | 5.025 | 5.825 | -0.8 | 0.616 | 12 | | 12 | 1.299 | | 297 | |
| BBL/L vs. BBL/D | 6.017 | 7.392 | -1.375 | 0.616 | 12 | | 12 | 2.232 | | 297 | |
| BBD/L vs. BBD/D | 7 | 7.608 | -0.6083 | 0.616 | 12 | | 12 | 0.9875 | | 297 | |
| BAB/L vs. BAB/D | 0.9583 | 2.383 | -1.425 | 0.616 | 12 | | 12 | 2.313 | | 297 | |
| BAL/L vs. BAL/D | 1.858 | 1.317 | 0.5417 | 0.616 | 12 | | 12 | 0.8793 | | 297 | |
| BAD/L vs. BAD/D | 3.642 | 1.958 | 1.683 | 0.616 | 12 | | 12 | 2.733 | | 297 | |

**SWEET**

Sweet preference differed significantly between SD/L and SD/D groups (F(27, 297) = 3.245, p < 0.0001, R² = 0.2278).

| Number of families | 1 |  |  |  |  |  |  |  |
| --- | --- | --- | --- | --- | --- | --- | --- | --- |
| Number of comparisons per family | 14 |  |  |  |  |  |  |  |
| Q | 0.1 |  |  |  |  |  |  |  |
| **Original FDR method of  Benjamini and Hochberg** | Mean Diff. | Discovery? | q value | Individual P Value | |  |  |  |
| D1BB/L vs. D1BB/D | -2.717 | **Yes** | **0.0466** | **0.0098** | M-N |  |  |  |
| D1BL/L vs. D1BL/D | -2.342 | Yes | 0.0899 | 0.0257 | O-P |  |  |  |
| D1BD/L vs. D1BD/D | -0.975 | No | 0.5464 | 0.3513 | Q-R |  |  |  |
| RBB/L vs. RBB/D | -2.708 | **Yes** | **0.0466** | **0.01** | Y-Z |  |  |  |
| D1AB/L vs. D1AB/D | -1.917 | No | 0.1889 | 0.0675 | S-T |  |  |  |
| D1AL/L vs. D1AL/D | -1.283 | No | 0.5136 | 0.2201 | U-V |  |  |  |
| D1AD/L vs. D1AD/D | -3.725 | **Yes** | **0.0059** | **0.0004** | W-X |  |  |  |
| RAB/L vs. RAB/D | -0.8417 | No | 0.5893 | 0.4209 | AA-AB |  |  |  |
| BBB/L vs. BBB/D | -0.075 | No | 0.9428 | 0.9428 | A-B |  |  |  |
| BBL/L vs. BBL/D | -0.625 | No | 0.6417 | 0.55 | C-D |  |  |  |
| BBD/L vs. BBD/D | -1.033 | No | 0.5464 | 0.3232 | E-F |  |  |  |
| BAB/L vs. BAB/D | -1.042 | No | 0.5464 | 0.3194 | G-H |  |  |  |
| BAL/L vs. BAL/D | 0.2 | No | 0.9135 | 0.8483 | I-J |  |  |  |
| BAD/L vs. BAD/D | -0.6667 | No | 0.6417 | 0.5237 | K-L |  |  |  |
| Test details | Mean 1 | Mean 2 | Mean Diff. | SE of diff. | n1 | n2 | t | DF |
| D1BB/L vs. D1BB/D | 2.15 | 4.867 | -2.717 | 1.044 | 12 | 12 | 2.601 | 297 |
| D1BL/L vs. D1BL/D | 4.508 | 6.85 | -2.342 | 1.044 | 12 | 12 | 2.242 | 297 |
| D1BD/L vs. D1BD/D | 5.642 | 6.617 | -0.975 | 1.044 | 12 | 12 | 0.9336 | 297 |
| RBB/L vs. RBB/D | 2.475 | 5.183 | -2.708 | 1.044 | 12 | 12 | 2.593 | 297 |
| D1AB/L vs. D1AB/D | 5.35 | 7.267 | -1.917 | 1.044 | 12 | 12 | 1.835 | 297 |
| D1AL/L vs. D1AL/D | 5.5 | 6.783 | -1.283 | 1.044 | 12 | 12 | 1.229 | 297 |
| D1AD/L vs. D1AD/D | 4.633 | 8.358 | -3.725 | 1.044 | 12 | 12 | 3.567 | 297 |
| RAB/L vs. RAB/D | 6.15 | 6.992 | -0.8417 | 1.044 | 12 | 12 | 0.8059 | 297 |
| BBB/L vs. BBB/D | 3.883 | 3.958 | -0.075 | 1.044 | 12 | 12 | 0.07181 | 297 |
| BBL/L vs. BBL/D | 5.058 | 5.683 | -0.625 | 1.044 | 12 | 12 | 0.5985 | 297 |
| BBD/L vs. BBD/D | 4.633 | 5.667 | -1.033 | 1.044 | 12 | 12 | 0.9895 | 297 |
| BAB/L vs. BAB/D | 4.717 | 5.758 | -1.042 | 1.044 | 12 | 12 | 0.9974 | 297 |
| BAL/L vs. BAL/D | 5.475 | 5.275 | 0.2 | 1.044 | 12 | 12 | 0.1915 | 297 |
| BAD/L vs. BAD/D | 4.958 | 5.625 | -0.6667 | 1.044 | 12 | 12 | 0.6384 | 297 |

| **Uncorrected Fisher's LSD** | Mean diff, | 95.00% CI of diff, | Below threshold? | Summary | Individual P Value |  |
| --- | --- | --- | --- | --- | --- | --- |
| D1BB/L vs. D1BB/D | -2.717 | -4.772 to -0.6614 | Yes | ** | 0.0098 |  |
| D1BL/L vs. D1BL/D | -2.342 | -4.397 to -0.2864 | Yes | * | 0.0257 |  |
| RBB/L vs. RBB/D | -2.708 | -4.764 to -0.6531 | Yes | ** | 0.0100 |  |
| D1AD/L vs. D1AD/D | -3.725 | -5.780 to -1.670 | Yes | *** | 0.0004 |  |

**SALTY**

Salty preference differed significantly between SD/L and SD/D groups (F(27, 297) = 5.590, p < 0.0001, R² = 0.3370).

| Number of families | 1 |  |  |  |  |  |  |  |
| --- | --- | --- | --- | --- | --- | --- | --- | --- |
| Number of comparisons per family | 14 |  |  |  |  |  |  |  |
| Q | 0.1 |  |  |  |  |  |  |  |
| **Original FDR method of  Benjamini and Hochberg** | Mean Diff. | Discovery? | q value | Individual  P Value | |  |  |  |
| D1BB/L vs. D1BB/D | -0.325 | No | 0.9722 | 0.7343 | M-N |  |  |  |
| D1BL/L vs. D1BL/D | 0.04167 | No | 0.9722 | 0.9653 | O-P |  |  |  |
| D1BD/L vs. D1BD/D | -0.075 | No | 0.9722 | 0.9376 | Q-R |  |  |  |
| RBB/L vs. RBB/D | 0.3333 | No | 0.9722 | 0.7277 | Y-Z |  |  |  |
| D1AB/L vs. D1AB/D | 1.05 | No | 0.765 | 0.2732 | S-T |  |  |  |
| D1AL/L vs. D1AL/D | 2.875 | **Yes** | **0.0231** | **0.0029** | U-V |  |  |  |
| D1AD/L vs. D1AD/D | 0.2667 | No | 0.9722 | 0.7806 | W-X |  |  |  |
| RAB/L vs. RAB/D | 0.6833 | No | 0.9722 | 0.4755 | AA-AB |  |  |  |
| BBB/L vs. BBB/D | -1.825 | No | 0.2008 | 0.0574 | A-B |  |  |  |
| BBL/L vs. BBL/D | 0.275 | No | 0.9722 | 0.7739 | C-D |  |  |  |
| BBD/L vs. BBD/D | 0.03333 | No | 0.9722 | 0.9722 | E-F |  |  |  |
| BAB/L vs. BAB/D | 2.833 | **Yes** | **0.0231** | **0.0033** | G-H |  |  |  |
| BAL/L vs. BAL/D | 0.2333 | No | 0.9722 | 0.8074 | I-J |  |  |  |
| BAD/L vs. BAD/D | -2.567 | **Yes** | **0.0359** | **0.0077** | K-L |  |  |  |
| Test details | Mean 1 | Mean 2 | Mean Diff. | SE of diff. | n1 | n2 | t | DF |
| D1BB/L vs. D1BB/D | 2.783 | 3.108 | -0.325 | 0.9565 | 12 | 12 | 0.3398 | 297 |
| D1BL/L vs. D1BL/D | 2.425 | 2.383 | 0.04167 | 0.9565 | 12 | 12 | 0.04356 | 297 |
| D1BD/L vs. D1BD/D | 2.867 | 2.942 | -0.075 | 0.9565 | 12 | 12 | 0.07841 | 297 |
| RBB/L vs. RBB/D | 4.108 | 3.775 | 0.3333 | 0.9565 | 12 | 12 | 0.3485 | 297 |
| D1AB/L vs. D1AB/D | 6.458 | 5.408 | 1.05 | 0.9565 | 12 | 12 | 1.098 | 297 |
| D1AL/L vs. D1AL/D | 7.375 | 4.5 | 2.875 | 0.9565 | 12 | 12 | 3.006 | 297 |
| D1AD/L vs. D1AD/D | 6.417 | 6.15 | 0.2667 | 0.9565 | 12 | 12 | 0.2788 | 297 |
| RAB/L vs. RAB/D | 4.508 | 3.825 | 0.6833 | 0.9565 | 12 | 12 | 0.7144 | 297 |
| BBB/L vs. BBB/D | 4 | 5.825 | -1.825 | 0.9565 | 12 | 12 | 1.908 | 297 |
| BBL/L vs. BBL/D | 4.508 | 4.233 | 0.275 | 0.9565 | 12 | 12 | 0.2875 | 297 |
| BBD/L vs. BBD/D | 3.183 | 3.15 | 0.03333 | 0.9565 | 12 | 12 | 0.03485 | 297 |
| BAB/L vs. BAB/D | 7.217 | 4.383 | 2.833 | 0.9565 | 12 | 12 | 2.962 | 297 |
| BAL/L vs. BAL/D | 6.958 | 6.725 | 0.2333 | 0.9565 | 12 | 12 | 0.2439 | 297 |
| BAD/L vs. BAD/D | 4.508 | 7.075 | -2.567 | 0.9565 | 12 | 12 | 2.683 | 297 |

| **Uncorrected Fisher's LSD** | Mean diff, | 95.00% CI of diff, | Below threshold? | Summary | Individual P Value |  |
| --- | --- | --- | --- | --- | --- | --- |
| D1AL/L vs. D1AL/D | 2.875 | 0.9926 to 4.757 | Yes | ** | 0.0029 |  |
| BAB/L vs. BAB/D | 2.833 | 0.9509 to 4.716 | Yes | ** | 0.0033 |  |
| BAD/L vs. BAD/D | -2.567 | -4.449 to -0.6842 | Yes | ** | 0.0077 |  |

**PICANT**

Picant preference differed significantly between SD/L and SD/D groups (F(27, 297) = 4.837, p < 0.0001, R² = 0.3054).

| Number of families | 1 |  |  |  |  |  |  |  |
| --- | --- | --- | --- | --- | --- | --- | --- | --- |
| Number of comparisons per family | 14 |  |  |  |  |  |  |  |
| Q | 0.1 |  |  |  |  |  |  |  |
| **Original FDR method of  Benjamini and Hochberg** | Mean Diff. | Discovery? | q value | Individual  P Value | |  |  |  |
| D1BB/L vs. D1BB/D | 0.575 | No | 0.8413 | 0.5408 | M-N |  |  |  |
| D1BL/L vs. D1BL/D | -0.1667 | No | 0.8942 | 0.8593 | O-P |  |  |  |
| D1BD/L vs. D1BD/D | 1.05 | No | 0.4829 | 0.2644 | Q-R |  |  |  |
| RBB/L vs. RBB/D | 3.708 | **Yes** | **0.0014** | **<0.0001** | Y-Z |  |  |  |
| D1AB/L vs. D1AB/D | 1.233 | No | 0.4436 | 0.1901 | S-T |  |  |  |
| D1AL/L vs. D1AL/D | 1.675 | No | 0.2505 | 0.0755 | U-V |  |  |  |
| D1AD/L vs. D1AD/D | -0.125 | No | 0.8942 | 0.8942 | W-X |  |  |  |
| RAB/L vs. RAB/D | 3.375 | **Yes** | **0.0027** | **0.0004** | AA-AB |  |  |  |
| BBB/L vs. BBB/D | -0.2333 | No | 0.8942 | 0.8039 | A-B |  |  |  |
| BBL/L vs. BBL/D | -0.25 | No | 0.8942 | 0.7903 | C-D |  |  |  |
| BBD/L vs. BBD/D | -1.025 | No | 0.4829 | 0.276 | E-F |  |  |  |
| BAB/L vs. BAB/D | 1.6 | No | 0.2505 | 0.0895 | G-H |  |  |  |
| BAL/L vs. BAL/D | -0.4917 | No | 0.8414 | 0.601 | I-J |  |  |  |
| BAD/L vs. BAD/D | -1.775 | No | 0.2505 | 0.0597 | K-L |  |  |  |
| Test details | Mean 1 | Mean 2 | Mean Diff. | SE of diff. | n1 | n2 | t | DF |
| D1BB/L vs. D1BB/D | 7.2 | 6.625 | 0.575 | 0.9391 | 12 | 12 | 0.6123 | 297 |
| D1BL/L vs. D1BL/D | 3.992 | 4.158 | -0.1667 | 0.9391 | 12 | 12 | 0.1775 | 297 |
| D1BD/L vs. D1BD/D | 4.458 | 3.408 | 1.05 | 0.9391 | 12 | 12 | 1.118 | 297 |
| RBB/L vs. RBB/D | 7.483 | 3.775 | 3.708 | 0.9391 | 12 | 12 | 3.949 | 297 |
| D1AB/L vs. D1AB/D | 7.5 | 6.267 | 1.233 | 0.9391 | 12 | 12 | 1.313 | 297 |
| D1AL/L vs. D1AL/D | 7.592 | 5.917 | 1.675 | 0.9391 | 12 | 12 | 1.784 | 297 |
| D1AD/L vs. D1AD/D | 6.842 | 6.967 | -0.125 | 0.9391 | 12 | 12 | 0.1331 | 297 |
| RAB/L vs. RAB/D | 7.2 | 3.825 | 3.375 | 0.9391 | 12 | 12 | 3.594 | 297 |
| BBB/L vs. BBB/D | 6.975 | 7.208 | -0.2333 | 0.9391 | 12 | 12 | 0.2485 | 297 |
| BBL/L vs. BBL/D | 4.767 | 5.017 | -0.25 | 0.9391 | 12 | 12 | 0.2662 | 297 |
| BBD/L vs. BBD/D | 4.075 | 5.1 | -1.025 | 0.9391 | 12 | 12 | 1.091 | 297 |
| BAB/L vs. BAB/D | 7.733 | 6.133 | 1.6 | 0.9391 | 12 | 12 | 1.704 | 297 |
| BAL/L vs. BAL/D | 7.283 | 7.775 | -0.4917 | 0.9391 | 12 | 12 | 0.5235 | 297 |
| BAD/L vs. BAD/D | 5.667 | 7.442 | -1.775 | 0.9391 | 12 | 12 | 1.89 | 297 |

| **Uncorrected Fisher's LSD** | Mean diff, | 95.00% CI of diff, | Below threshold? | Summary | Individual P Value |  |
| --- | --- | --- | --- | --- | --- | --- |
| RBB/L vs. RBB/D | 3.708 | 1.860 to 5.556 | Yes | **** | <0.0001 |  |
| RAB/L vs. RAB/D | 3.375 | 1.527 to 5.223 | Yes | *** | 0.0004 |  |

**GREASY**

Greasy preference differed significantly between SD/L and SD/D groups (F(27, 297) = 5.355, p < 0.0001, R² = 0.3274).

| Number of families | 1 |  |  |  |  |  |  |  |
| --- | --- | --- | --- | --- | --- | --- | --- | --- |
| Number of comparisons per family | 14 |  |  |  |  |  |  |  |
| Q | 0.1 |  |  |  |  |  |  |  |
| **Original FDR method of  Benjamini and Hochberg** | Mean Diff. | Discovery? | q value | Individual P Value | |  |  |  |
| D1BB/L vs. D1BB/D | 0.1 | No | 0.9064 | 0.9064 | M-N |  |  |  |
| D1BL/L vs. D1BL/D | 0.9917 | No | 0.8544 | 0.2441 | O-P |  |  |  |
| D1BD/L vs. D1BD/D | 0.4417 | No | 0.9064 | 0.6036 | Q-R |  |  |  |
| RBB/L vs. RBB/D | -0.225 | No | 0.9064 | 0.7913 | Y-Z |  |  |  |
| D1AB/L vs. D1AB/D | -0.4667 | No | 0.9064 | 0.5833 | S-T |  |  |  |
| D1AL/L vs. D1AL/D | 0.85 | No | 0.8903 | 0.318 | U-V |  |  |  |
| D1AD/L vs. D1AD/D | -0.2583 | No | 0.9064 | 0.7613 | W-X |  |  |  |
| RAB/L vs. RAB/D | 0.1667 | No | 0.9064 | 0.8446 | AA-AB |  |  |  |
| BBB/L vs. BBB/D | -0.2583 | No | 0.9064 | 0.7613 | A-B |  |  |  |
| BBL/L vs. BBL/D | 1.125 | No | 0.8544 | 0.1865 | C-D |  |  |  |
| BBD/L vs. BBD/D | -0.3333 | No | 0.9064 | 0.6951 | E-F |  |  |  |
| BAB/L vs. BAB/D | 2.092 | No | 0.2016 | 0.0144 | G-H |  |  |  |
| BAL/L vs. BAL/D | 0.5917 | No | 0.9064 | 0.4868 | I-J |  |  |  |
| BAD/L vs. BAD/D | -1.292 | No | 0.8544 | 0.1295 | K-L |  |  |  |
| Test details | Mean 1 | Mean 2 | Mean Diff. | SE of diff. | n1 | n2 | t | DF |
| D1BB/L vs. D1BB/D | 5.083 | 4.983 | 0.1 | 0.8497 | 12 | 12 | 0.1177 | 297 |
| D1BL/L vs. D1BL/D | 4.825 | 3.833 | 0.9917 | 0.8497 | 12 | 12 | 1.167 | 297 |
| D1BD/L vs. D1BD/D | 4.8 | 4.358 | 0.4417 | 0.8497 | 12 | 12 | 0.5198 | 297 |
| RBB/L vs. RBB/D | 5.642 | 5.867 | -0.225 | 0.8497 | 12 | 12 | 0.2648 | 297 |
| D1AB/L vs. D1AB/D | 7.367 | 7.833 | -0.4667 | 0.8497 | 12 | 12 | 0.5492 | 297 |
| D1AL/L vs. D1AL/D | 8.4 | 7.55 | 0.85 | 0.8497 | 12 | 12 | 1 | 297 |
| D1AD/L vs. D1AD/D | 7.533 | 7.792 | -0.2583 | 0.8497 | 12 | 12 | 0.304 | 297 |
| RAB/L vs. RAB/D | 6.633 | 6.467 | 0.1667 | 0.8497 | 12 | 12 | 0.1961 | 297 |
| BBB/L vs. BBB/D | 5.908 | 6.167 | -0.2583 | 0.8497 | 12 | 12 | 0.304 | 297 |
| BBL/L vs. BBL/D | 5.367 | 4.242 | 1.125 | 0.8497 | 12 | 12 | 1.324 | 297 |
| BBD/L vs. BBD/D | 4.017 | 4.35 | -0.3333 | 0.8497 | 12 | 12 | 0.3923 | 297 |
| BAB/L vs. BAB/D | 7.692 | 5.6 | 2.092 | 0.8497 | 12 | 12 | 2.462 | 297 |
| BAL/L vs. BAL/D | 7.933 | 7.342 | 0.5917 | 0.8497 | 12 | 12 | 0.6963 | 297 |
| BAD/L vs. BAD/D | 6.117 | 7.408 | -1.292 | 0.8497 | 12 | 12 | 1.52 | 297 |


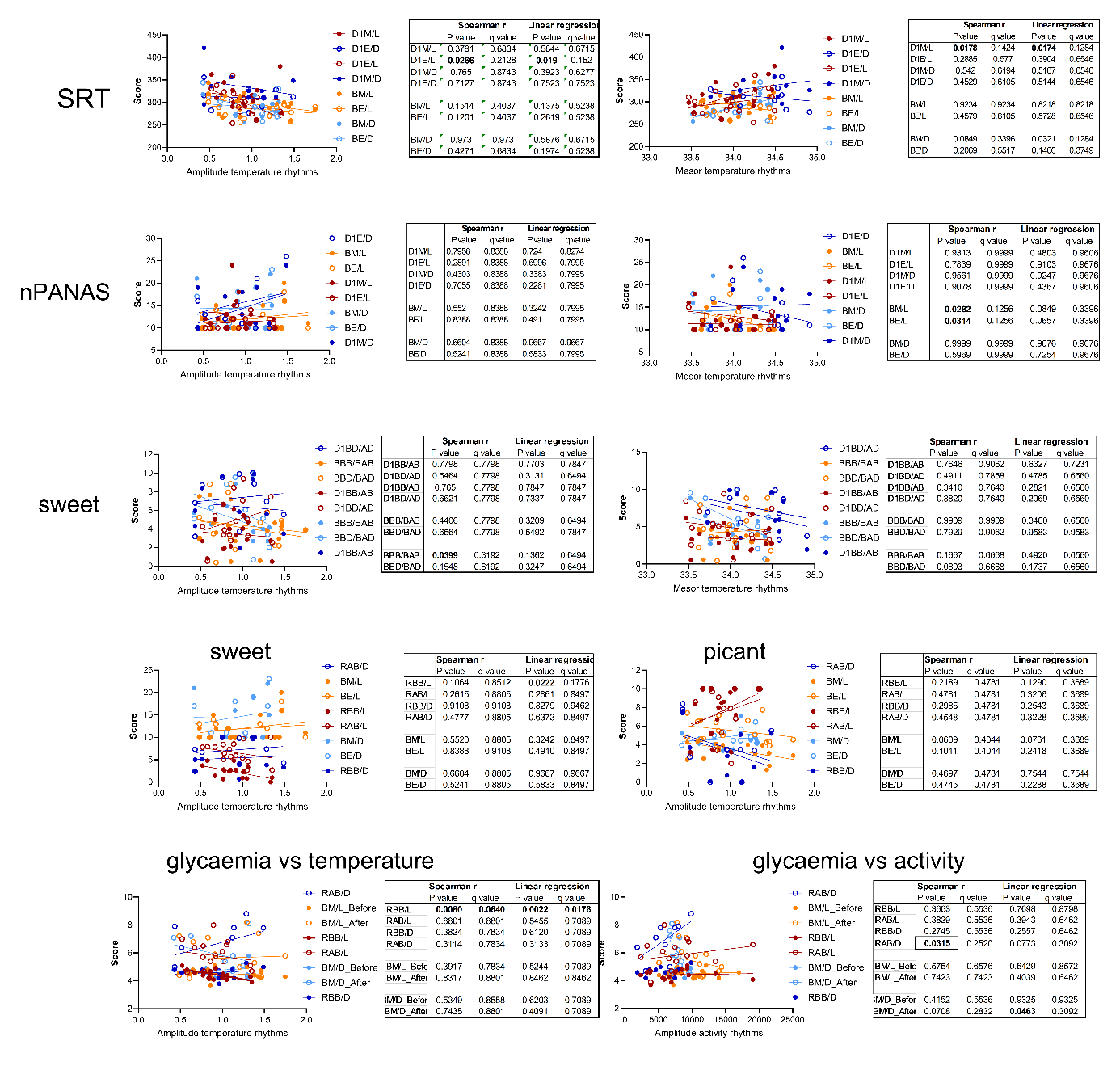


**Figure S1.** **Spearman correlations** were calculated between the amplitude and mesor of the circadian rhythm in body temperature and parameters significantly altered between the SD/L and SD/D groups. In addition to summary figures, a table presents significance values: the first column shows p-values for Spearman correlations, and the third column shows results from simple linear correlations testing whether the slope differs significantly from zero. The second and fourth columns show corresponding values corrected using Benjamini and Hochberg’s False Discovery Rate (FDR) method.

The results suggest, for example, that SRT performance may improve with increasing amplitude or decreasing mesor of the rhythm, or that negative PANAS scores may relate to the mesor of body temperature in certain groups. However, none of these associations are supported by FDR correction, suggesting they are likely due to chance. Similar patterns were found for amplitude and mesor of the rhythm in physical activity. An exception is fasting glycaemia on the recovery day: in the SD/L group, a significant correlation with the amplitude of body temperature rhythm was observed and remained after FDR correction. This association is highlighted with a bold line in the figure. However, interpretation remains difficult, as it is not supported by a corresponding correlation with the amplitude of the activity rhythm.
